# Natural Killer Cell Sensing of Infected Cells Compensates for MyD88 Deficiency but Not IFN-I Activity in Resistance to Mouse Cytomegalovirus

**DOI:** 10.1371/journal.ppat.1004897

**Published:** 2015-05-08

**Authors:** Clément Cocita, Rachel Guiton, Gilles Bessou, Lionel Chasson, Marilyn Boyron, Karine Crozat, Marc Dalod

**Affiliations:** 1 Centre d’Immunologie de Marseille-Luminy, UNIV UM2, Aix Marseille Université, Parc Scientifique et Technologique de Luminy, Marseille, France; 2 Institut National de la Santé et de la Recherche Médicale (INSERM), U1104, Marseille, France; 3 Centre National de la Recherche Scientifique (CNRS), UMR7280, Marseille, France; University of California, San Francisco, UNITED STATES

## Abstract

In mice, plasmacytoid dendritic cells (pDC) and natural killer (NK) cells both contribute to resistance to systemic infections with herpes viruses including mouse Cytomegalovirus (MCMV). pDCs are the major source of type I IFN (IFN-I) during MCMV infection. This response requires pDC-intrinsic MyD88-dependent signaling by Toll-Like Receptors 7 and 9. Provided that they express appropriate recognition receptors such as Ly49H, NK cells can directly sense and kill MCMV-infected cells. The loss of any one of these responses increases susceptibility to infection. However, the relative importance of these antiviral immune responses and how they are related remain unclear. In humans, while IFN-I responses are essential, MyD88 is dispensable for antiviral immunity. Hence, a higher redundancy has been proposed in the mechanisms promoting protective immune responses against systemic infections by herpes viruses during natural infections in humans. It has been assumed, but not proven, that mice fail to mount protective MyD88-independent IFN-I responses. In humans, the mechanism that compensates MyD88 deficiency has not been elucidated. To address these issues, we compared resistance to MCMV infection and immune responses between mouse strains deficient for MyD88, the IFN-I receptor and/or Ly49H. We show that selective depletion of pDC or genetic deficiencies for MyD88 or TLR9 drastically decreased production of IFN-I, but not the protective antiviral responses. Moreover, MyD88, but not IFN-I receptor, deficiency could largely be compensated by Ly49H-mediated antiviral NK cell responses. Thus, contrary to the current dogma but consistent with the situation in humans, we conclude that, in mice, in our experimental settings, MyD88 is redundant for IFN-I responses and overall defense against a systemic herpes virus infection. Moreover, we identified direct NK cell sensing of infected cells as one mechanism able to compensate for MyD88 deficiency in mice. Similar mechanisms likely contribute to protect MyD88- or IRAK4-deficient patients from viral infections.

## Author Summary

Type I interferons (IFN-I) are innate cytokines crucial for vertebrate antiviral defenses. IFN-I exert antiviral effector functions and orchestrate antiviral immunity. IFN-I are induced early after infection, upon sensing of viral particles or infected cells by immune receptors. Intracellular Toll-like receptors (TLR) are selectively expressed in specialized immune cell types such as plasmacytoid dendritic cells (pDC), enabling them to copiously produce IFN-I upon detection of engulfed viral nucleic acids. pDC or intracellular TLR have been reported to be crucial for resistance to experimental infections with many viruses in mice but dispensable for resistance to natural infections in humans. Our aim was to investigate this puzzling difference. Mice deficient for TLR activity mounted strong IFN-I responses despite producing very low IFN-I levels and controlled the infection by a moderate dose of murine cytomegalovirus much better than mice deficient for IFN-I responses. Deficient TLR responses could be compensated by direct recognition of infected cells by natural killer cells. Hence, we identified experimental conditions in mice mimicking the lack of requirement of TLR functions for antiviral defense observed in humans. We used these experimental models to advance our basic understanding of antiviral immunity in a way that might help improve treatments for patients.

## Introduction

Type I interferons (IFN-I) orchestrate vertebrate antiviral defenses through two complementary mechanisms [1]. These cytokines induce multiple Interferon Stimulated Genes (ISG) coding for effector molecules of cell-intrinsic antiviral immunity. IFN-I instruct antiviral innate and adaptive immunity, in part by promoting the maturation of dendritic cells (DC) for potent activation of natural killer (NK) cells and CD8 T lymphocytes. Genetic deficiencies compromising IFN-I responses dramatically increase susceptibility to viral infections in mice and men [2]. In addition to IFN-I, type III interferons (IFN-III) also appear critical for antiviral defense, based on the analysis of mutant mice, and on the strong association between resistance to viral infections and polymorphisms affecting these genes in humans [1, 3, 4]. IFN-I and IFN-III share the same signaling pathways and downstream target genes. However, while the IFN-I receptor (IFNAR) is ubiquitously expressed, the receptor for IFN-III is selectively expressed in epithelial cells [1].

The contribution of different cell types and molecular sensors to IFN-I induction during viral infections is the subject of debate. IFN-I can be induced by two major mechanisms in infected hosts [1]. Potentially all host cell types are equipped with innate immune sensors of endogenous viral replication that can trigger IFN-I production. Certain immune cell types are also able to sense viral infection in their surroundings and consequently produce high levels of IFN-I without being infected. This ability is especially strong in the plasmacytoid subset of DC (pDC). pDC recognize and engulf viral particles or material derived from infected cells. Subsequent detection of nucleic acids by toll-like receptors (TLR) 7 and 9 in specialized endosomes leads to MyD88- and IRAK4-dependent IFN-I induction [5]. On the other hand, while patients genetically deficient for *MYD88* or *IRAK4* show enhanced susceptibility to mycobacteria, they are resistant to most viral infections [6–8]. MyD88 is critical for signaling not only by all TLRs except TLR3 but also by the receptors for all members of the interleukin-1 (IL-1) cytokine family [9]. Hence, in humans, pDC, TLR7/8/9 and all the IL-1 cytokine family are largely redundant for antiviral defense, in particular with regards to induction of protective IFN-I responses [8]. In contrast, mice genetically deficient for *Tlr7*, *Tlr9* or *Myd88* show enhanced susceptibility to a broad range of pathogens including many viruses [8]. pDC production of IFN-I has been proposed to be essential in mice for the control of acute systemic viral infections in particular with herpes viruses [10–12] or coronaviruses [13]. pDC may also contribute to prevent the establishment of chronic infections with certain viruses [14–16]. Here, we designed experimental studies to investigate what could explain this reported discrepancy between humans and mice for the importance of MyD88 responses in antiviral defense. During viral infections in mice, the impact of MyD88 inactivation or pDC depletion had only been assessed on the basis of IFN-I production. Here, we also examined how it affected the induction of protective IFN-I responses. We also investigated whether NK cell responses could compensate for MyD88 deficiency for host resistance.

We used experimental infection by murine cytomegalovirus (MCMV), a natural pathogen of mice [17], for which IFN-I responses are critical for protection both *in vitro* in macrophages [18–21] and *in vivo* [22, 23]. pDC sense MCMV infection through TLR7/9 [24–27] and constitute the major source of IFN-I *in vivo* [10, 24, 28, 29]. However, infected stromal cells have also been described as potent IFN-I producers in the spleen, 8 hrs after infection [30]. NK cells sense MCMV infection through their activating receptor Ly49H, allowing them to specifically recognize and kill infected cells through binding to m157, a viral protein expressed at their surface. pDC depletion prior to infection leads to a dramatic decrease of serum IFN-I levels but only to a modest and transient increase in viral loads in most organs [5, 10, 24, 29]. In contrast, either NK cell depletion, or deficiency in endosomal TLR activity, decreases the ability of C57BL/6 mice to control viral replication and increases morbidity and mortality [24–27, 31]. It is assumed that the enhanced susceptibility to certain systemic viral infections of mice deficient for endosomal TLR activity is largely due to their decreased IFN-I production [8, 10–13, 24, 27]. However, whether tissue responses to IFN-I are reduced in these animals has not been examined. Moreover, other immune functions are dampened or lost in these animals, including IL-12 production [5] and, in MyD88-deficient mice, responses to all member of the IL-1 cytokine family [9]. Moreover, the respective contributions of IFN-I-, MyD88- and Ly49H-dependent responses to overall resistance to MCMV infection has not been rigorously investigated in parallel in mice of the same genetic background, deficient for one or more of these responses. We thus designed a study to rigorously explore how MyD88 genetic deficiency in mice affects their responses to MCMV infection, in particular their ability to mount strong type I IFN responses and to resist disease development, for comparison with the analysis previously published in patients genetically deficient for *MYD88* [7]. In both species, MyD88 deficiency does not only affect direct viral sensing by TLRs but also signaling by all IL-1 family cytokines including IL-18. We also wanted to explore whether the consequences of MyD88 deficiency could be modulated by mutations or polymorphisms in other immune genes, for example those encoding NK cell activating receptors. In steady state conditions, in mice, IL-18 has been reported to be crucial for NK cell functional maturation, enabling them to respond to activation with synthetic stimuli [32]. Hence, using MyD88-deficient animals to examine the interaction between DC and NK cell direct sensing of infected cells might not seem optimal since MyD88-deficiency could affect NK cell responses in a cell-intrinsic manner. During MCMV infection *in vivo*, contradictory results have been reported regarding the role of IL-18 in the promotion of the proliferation of Ly49H^+^ NK cells, with no role observed in one study [33], an absolute requirement reported in a another study [34], and an important contribution but not an absolute requirement in a third study [35]. In any case, IL-18 has been described as a cytokine crucial for IFN-γ production by NK cells in the spleen but not in the liver and appears to be dispensable for resistance to MCMV infection under conditions where IL-12 and NK cells are critical [36]. In other words, IL-18 does not seem to be required for NK cell-dependent protection against primary MCMV infection. CD8 T cell responses are also important for immune defense against CMV infection in human and mice [5]. TLR or IL-1 stimulation of DC are considered to be critical for the induction of protective antiviral cellular adaptive immunity [37, 38]. CD8 T cell responses against MCMV are altered in mice affected in their MyD88 or NK cell activities [24, 39–42]. However, how NK and MyD88 responses are integrated in the shaping of antiviral CD8 T cells responses has not been examined.

We used a series of mutant BALB/c mice expressing or not Ly49H, and deficient or not for MyD88 or for the receptor for IFNAR. We characterized their immune response and overall resistance to MCMV infection. We used BALB/c congenic animals, C.B6-Klra8Cmv1-r/UwaJ mice referred to as BALB/c-Ly49H^+^ mice in the manuscript, which carry most of the C57BL/6 NK gene complex, not only limited to *Ly49h* [43]. Contrary to the situation observed in BALB/c mice, in BALB/c-Ly49H^+^ mice NK cells play a major role in the control of an *in vitro* grown WT MCMV Smith virus. This protective NK cell activity is almost completely abrogated when using an *in vitro* grown MCMV Smith virus strain lacking the *m157* gene [44]. Here, we compared viral control and mortality between BALB/c and BALB/c-Ly49H^+^ mice upon infection with a K181 virus strain lacking the *m157* gene, Δm157 MCMV [45]. Upon infection with a moderate dose of salivary gland-extracted Δm157 MCMV, BALB/c-Ly49H^+^ mice did not control viral replication more efficiently or survive better than BALB/c animals (S1A–S1C Fig). Altogether, these observations strongly suggest that most of the differences observed in the increased resistance to MCMV infection of BALB/c-Ly49H^+^ mice as compared to BALB/c animal likely result from the much more efficient recognition of infected cells by their NK cells, due to the interaction of the Ly49H NK cell activation receptor with the viral m157 protein expressed at the surface of infected cells. Nevertheless, some contributions for other genes encoded in the NK gene complex cannot be totally excluded. We chose to inject MCMV intraperitoneally because it is the most frequent route of inoculation used with MCMV and because it rapidly causes a systemic infection, which resolution is thought to more stringently depend of systemic production of IFN-I by pDC than for local infections. Indeed, with several other viral infections, including infections with the herpesviruses HSV-1 and HSV-2, pDC were shown to contribute to IFN-I production only under condition of systemic viral spread and not under conditions where the infections is performed and controlled in epithelia [12, 46]. Moreover, in mice, pDC depletion compromises the control of HSV-1 or HSV-2 only for systemic but not local infections [12]. Our results demonstrate that, in our experimental settings, MyD88 but not IFN-I responses are largely redundant for control of a systemic herpes virus infection in mice. This contradicts current thinking but reconciles the requisite of mice and men for innate antiviral defense. We identify direct recognition of infected cells by NK cells as one innate immune sensing mechanism able to compensate in part for the loss of MyD88 activity. Our results also highlight an unexpected ability of low levels of IFN-I to induce strong cell-intrinsic antiviral immunity *in vivo*, emphasizing the importance of measuring responses to, rather than production of, cytokines to assess their physiological role.

## Results

### pDC or MyD88 deficiency dramatically decreases IFN-I production but still allows a strong ISG induction during MCMV infection

**Fig 1:**
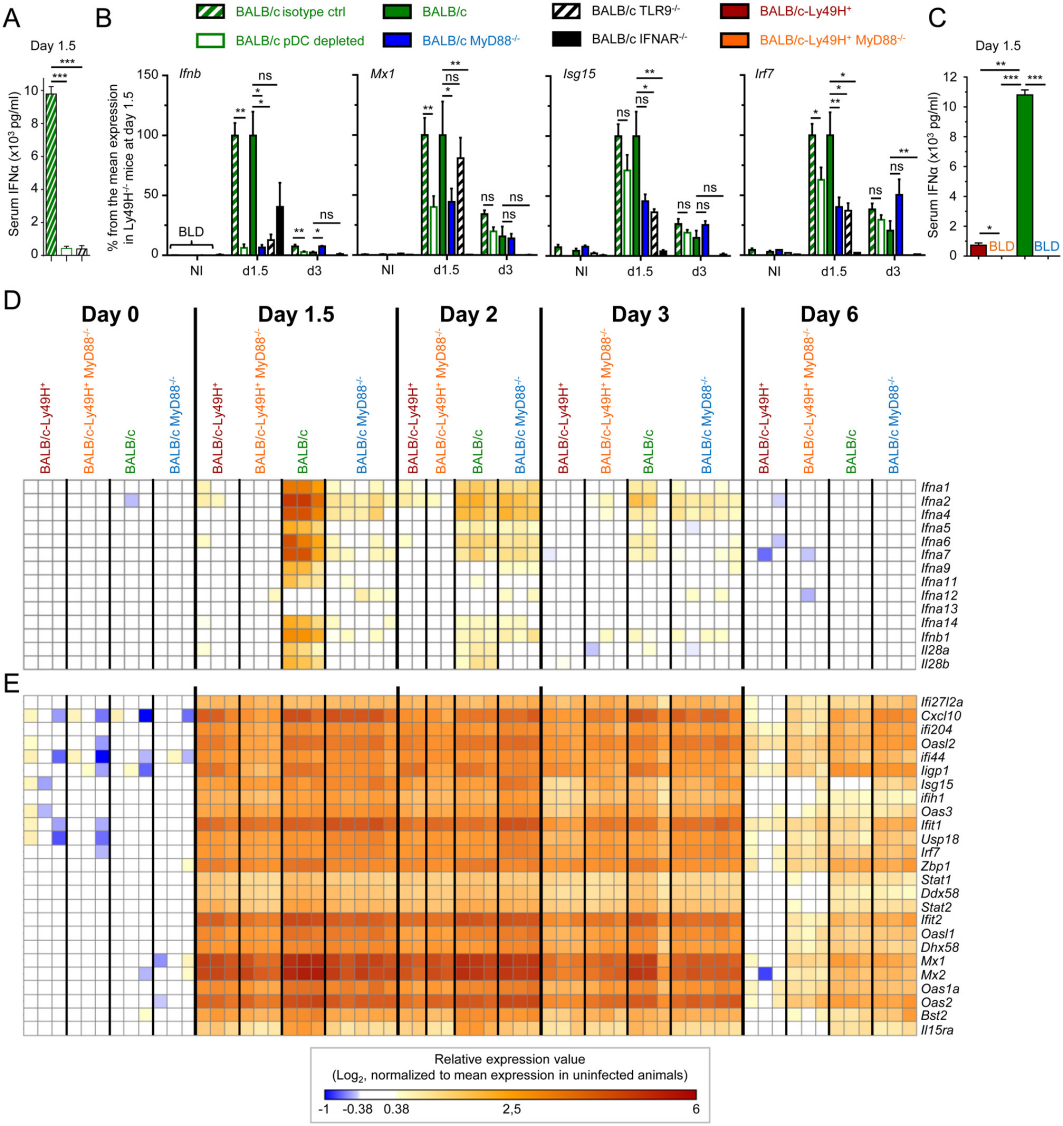
pDC or MyD88 deficiency impairs IFN-I production but not splenic IFN-I responses and virus control. Control (Rat IgG), pDC-depleted (αBST2) or untreated BALB/c mice and untreated BALB/c, BALB/c MyD88^-/-^, BALB/c IFNAR^-/-^ and BALB/c TLR9^-/-^ mice were infected with 2.5 x 10^3^ pfu MCMV or left uninfected (NI). (A) IFN-α serum titers were measured at d1.5 post-infection by ELISA. Results (mean±SEM) are shown from one experiment representative of 3 independent ones. (B) RT-PCR analysis of the expression of selected genes in the spleen at d1.5 or 3 post-infection. Data are normalized to mean expression of d1.5 BALB/c mice (100% reference level). Results (mean±SEM) are shown from 2 pooled independent experiments, each with 2 to 3 mice per group. (C) Serum IFN-α levels were measured at d1.5 post-infection by ELISA in BALB/c-Ly49H^+^, BALB/c-Ly49H^+^ MyD88^-/-^, BALB/c and BALB/c MyD88^-/-^ mice. Results (mean±SEM) are shown from one experiment representative of 3 independent ones, each with 3 mice per group. BLD: below limit of detection. (D-E) Microarray analyses were performed on total mRNA extracted from spleen at d0, 1.5, 2, 3 and 6 post-infection. Heatmaps show the relative expression value for IFN-I/III genes (D) and for 25 ISG (E). The expression pattern of ISG is examined globally in S2E–S2G Fig. Results shown are from 2 pooled independent experiments, each with 1 to 3 mice per group.

To assess the contribution of pDC activation and endosomal TLR7/9 triggering to IFN-I responses during MCMV infection, we analyzed the induction of IFN-I and ISG in the spleen or blood of BALB/c mice knocked-out for MyD88 or TLR9, or depleted of pDC through administration of the 120G8 mAb directed against Bst2. 120G8 mAb injection dramatically reduced circulating IFN-I titers and splenic *Ifnb1* expression at d1.5 after infection (Fig 1A and 1B). However, unexpectedly, only a slight impairment was observed in splenic induction of 3 canonical ISG at d1.5 and 3 after infection (Fig 1B). Consistently, 120G8 mAb injection in BALB/c mice did not compromise splenic viral control at d6 (S2A and S2B Fig). Because the administration of the 120G8 mAb resulted in an efficient and selective, even though not entirely specific, depletion of pDC during MCMV infection (see materials and methods), we can rigorously conclude that pDC and their high systemic production of type I IFN are not required for induction of strong type I IFN responses and relatively efficient control of MCMV infection in our experimental settings. Similar results were obtained for splenic induction of *Ifnb1* and ISG in BALB/c MyD88^-/-^ and BALB/c TLR9^-/-^ mice (Fig 1B and S2C Fig). In contrast, ISG induction was completely abrogated in IFNAR^-/-^ animals (Fig 1B). Thus induction of strong IFN-I responses in the spleen of MCMV infected mice seems not to require pDC, MyD88 or TLR9 functions and can be induced by very low levels of IFN-I.

### High IFN-I responses are observed in MCMV-infected mice, irrespective of pDC activation and IFN-I serum titers

Systemic levels of IFN-I during MCMV infection are controlled by a balance between pDC and NK cell activation [5]. High systemic IFN-I production not only requires the ability of pDC to sense viral nucleic acids through functional endosomal TLR, but is also promoted by viral replication, which is normally limited by NK cell activity. Hence, to generalize our observation that very low levels of IFN-I production are sufficient to induce strong IFN-I responses, we next measured pangenomic ISG induction in mice with low serum IFN-I titers (Fig 1C) and splenic pDC IFN-I expression (S2A and S2D Fig) resulting either from a primary immune deficiency (MyD88^-/-^ animals) or on the contrary from early control of the virus by NK cells (Ly49H^+^ animals). A strong induction of most IFN-I/III genes was observed in BALB/c mice at d1.5 after MCMV infection. This progressively decreased over time to become undetectable by d6 (Fig 1D). In contrast, hardly any induction of IFN-I/III genes was seen in BALB/c-Ly49H^+^ and BALB/c-Ly49H^+^ MyD88^-/-^ animals, and only a weaker and delayed induction was observed in BALB/c MyD88^-/-^ mice (Fig 1D). However, strikingly, a very strong ISG induction was observed in all mouse strains, already at d1.5 after MCMV infection. This was maintained until d3 in all mouse strains and was still strong at d6 in BALB/c and BALB/c MyD88^-/-^ mice (Fig 1E and S2E–S2G Fig). Thus, induction of strong IFN-I responses in the spleen of MCMV infected mice does not require pDC, MyD88 and TLR9 function and can be induced optimally under conditions where IFN-I/III are undetectable not only in the circulation but also in the spleen both at protein and mRNA levels. Hence, contrary to the commonly accepted dogma, pDC, MyD88 and TLR9 are dispensable in mice for induction of strong IFN-I responses against systemic infection with a herpes virus, in our experimental settings.

### IFN-I responses are essential to promote resistance to MCMV infection irrespective of Ly49H expression and confer a significant level of protection even in MyD88^-/-^ deficient animals

**Fig 2:**
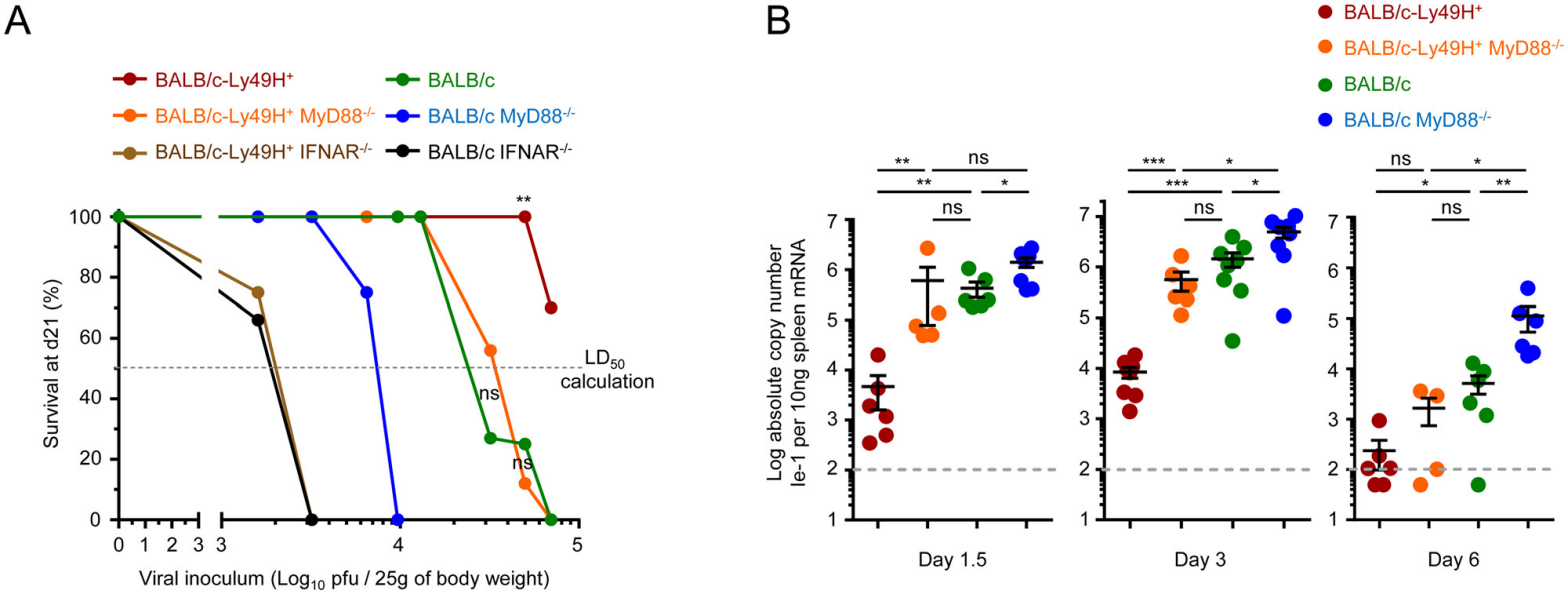
IFN-I responses are essential but MyD88 and Ly49H partly redundant to protect against MCMV infection. (A) Survival of mice at d21 post-infection (Y-axis) as a function of the doses of MCMV inoculum (X-axis). Indicated mice were infected with between 1.6x10^3^ and 7x10^4^ PFU of MCMV, with overlaps for several doses between strains of mice, and their survival was followed for 21d. Data is shown as percentage of survival. In total, 8 different doses were tested in 9 different experiments in order to determine the susceptibility of each mouse strain, with 2 to 4 mouse strains simultaneously studied in each experiment. Data was derived from the following numbers of mice and tested doses of MCMV inoculum: BALB/c-Ly49H^+^: 24 mice for 4 doses; BALB/c-Ly49H^+^ MyD88^-/-^: 37 mice for 6 doses; BALB/c-Ly49H^+^ IFNAR^-/-^: 8 mice for 2 doses; BALB/c: 43 mice for 5 doses; BALB/c MyD88^-/-^: 15 mice for 4 doses; BALB/c IFNAR^-/-^: 6 mice for 2 doses. The median lethal dose that causes 50% of lethality in each mouse strain (LD_50_) was calculated from the graph as the dose on the X-axis that corresponds to 50% mortality on the Y-axis as represented by the horizontal dotted line labeled “LD_50_ calculation”. LD_50_ BALB/c-Ly49H^+^: ≥10^5^ pfu/mouse; LD_50_ BALB/c-Ly49H^+^ MyD88^-/-^: 3.3x10^4^ pfu; LD_50_ BALB/c-Ly49H^+^ IFNAR^-/-^: 2x10^3^ pfu; LD_50_ BALB/c: 2.2x10^4^ pfu; LD_50_ BALB/c MyD88^-/-^: 8x10^3^ pfu; LD_50_ BALB/c IFNAR^-/-^: 2x10^3^ pfu. (B) Splenic viral titers were measured at d1.5, 3 and 6 post-infection. Data (mean±SEM) are shown from 2 to 4 pooled independent experiments, each with 2 to 3 mice per group.

We next examined to what extent IFN-I responses are required for defense against MCMV under our experimental conditions. We tested susceptibility to MCMV infection of 6 different mouse strains, with deficiencies in either IFNAR or MyD88, and expressing or not Ly49H (Fig 2), or selectively depleted for pDC (S3 Fig). We infected these animals with serial doses of MCMV, to define the LD_50_ inoculum at which 50% of the animals were killed (Fig 2A). As expected, BALB/c-Ly49H^+^ mice were the most resistant to infection. BALB/c IFNAR^-/-^ and BALB/c-Ly49H^+^ IFNAR^-/-^ mice were the most susceptible, with an LD_50_ more than 50 times lower than that of BALB/c-Ly49H^+^ animals. Thus, IFN-I responses are critical for defense against MCMV infection even in mice bearing the Ly49H activating receptor allowing direct sensing of infected cells by NK cells. Strikingly, BALB/c-Ly49H^+^ MyD88^-/-^ and BALB/c MyD88^-/-^ mice harbored clearly higher LD_50_ than IFNAR^-/-^ animals. Hence, MyD88^-/-^ mice were significantly more resistant than IFNAR^-/-^ animals, despite their low to undetectable production of IFN-I. In addition, pDC depletion did not increase the susceptibility of BALB/c mice after infection with the LD_50_ (S3 Fig). Thus, the induction of IFN-I responses to levels allowing a significant control of MCMV infection does not require MyD88 and pDC functions.

### Contrary to IFNAR deficiency, MyD88 deficiency can be compensated at least in part by direct sensing of infected cells by NK cells

**Fig 3:**
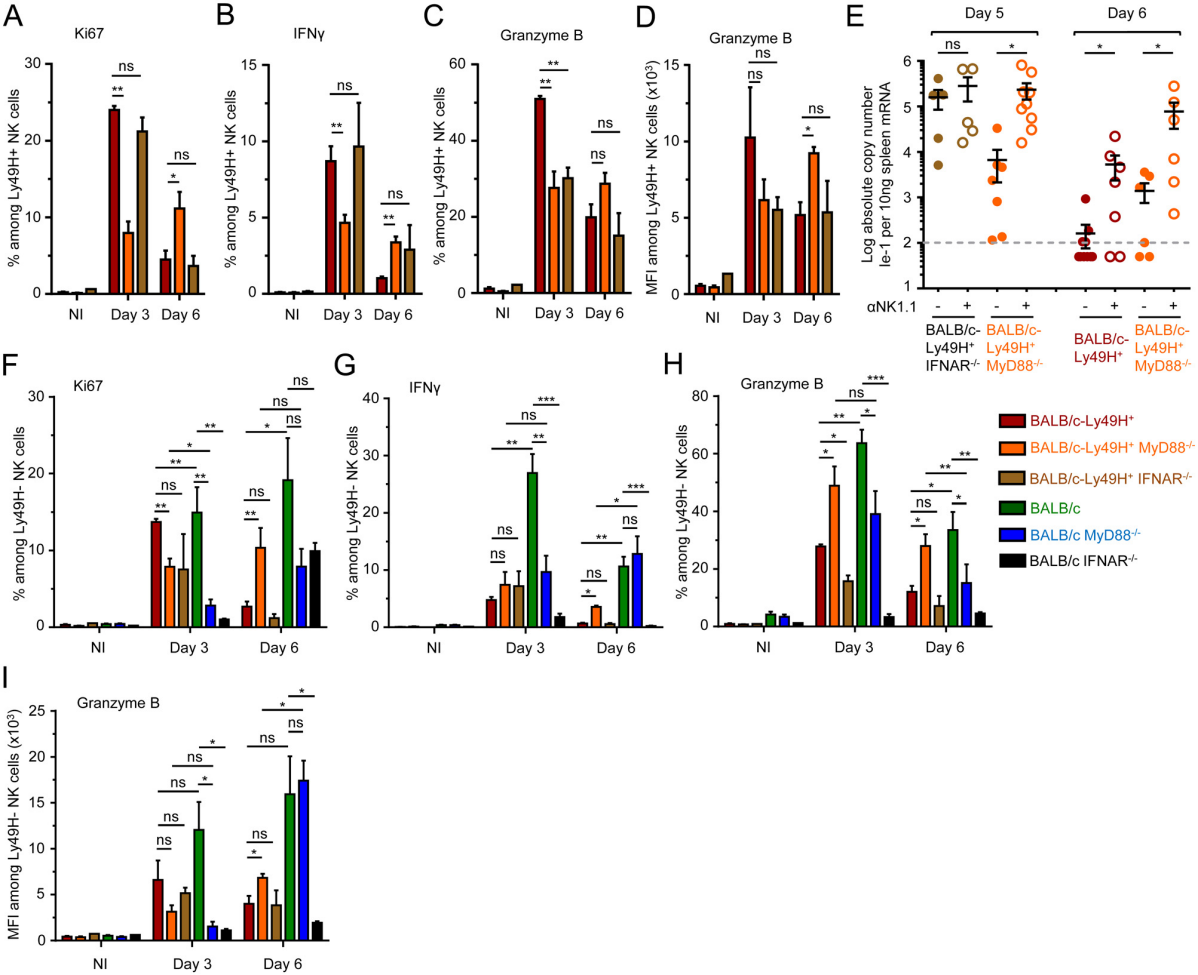
MyD88 deficiency does not completely abrogate NK cell-mediated protection. (A-D) Ly49H^+^ NK cell activation in BALB/c-Ly49H^+^, BALB/c-Ly49H^+^ MyD88^-/-^ and BALB/c-Ly49H^+^ IFNAR^-/-^ mice at d0, 3 and 6 post infection. Proliferation was assessed by Ki67 expression (A). Antiviral effector functions were assessed by intracellular staining for IFN-γ (B) and Granzyme B (C-D). (E) Impact of NK cell depletion on viral replication in the spleen of BALB/c-Ly49H^+^, BALB/c-Ly49H^+^ MyD88^-/-^ and BALB/c-Ly49H^+^ IFNAR^-/-^ mice. Mice were depleted of NK cells and splenic viral titers were measured 5 or 6d post infection. (F-I) Ly49H^-^ NK cell activation in BALB/c-Ly49H^+^, BALB/c-Ly49H^+^ MyD88, BALB/c-Ly49H^+^ IFNAR^-/-^, BALB/c, BALB/c MyD88^-/-^ and BALB/c IFNAR^-/-^ mice. Data (mean±SEM) are shown from 3 pooled independent experiments each with 3 mice per group.

The sensitivity to MCMV infection conferred by IFNAR deficiency was independent of the expression of Ly49H. This was not the case for MyD88 deficiency. BALB/c-Ly49H^+^ MyD88^-/-^ mice were clearly more resistant than BALB/c MyD88^-/-^ mice (Fig 2A). Conversely, the sensitivity to MCMV infection associated with Ly49H deficiency was aggravated by MyD88 deficiency, since BALB/c mice were more resistant than BALB/c MyD88^-/-^ mice (Fig 2A). BALB/c and BALB/c-Ly49H^+^ MyD88^-/-^ mice exhibited similar LD_50_. Hence, the deficiency in TLR-mediated MCMV sensing by DC and in responses to IL-1 cytokine family can be compensated at least in part by direct sensing of infected cells by NK cells and *vice versa*. Indeed, under conditions of infection with a moderate MCMV dose, below the LD_50_ of BALB/c MyD88^-/-^ mice, control of viral replication was significantly less efficient in these animals than in the three other mouse strains examined (Fig 2B). NK cell activation during MCMV infection depends on IL-12, IL-18 and IL-15 [5, 35]. The induction and/or activity of IL-12 and IL-18 are both strongly decreased in MyD88^-/-^ C57BL/6 mice. This correlates with a significant impairment of NK cell responses under conditions of high viral inoculum [5]. In contrast, here, the survival and viral titers observed suggested that functional antiviral NK cell responses were induced in BALB/c-Ly49H^+^ MyD88^-/-^ mice. Thus, we examined NK cell functions in BALB/c mice deficient or competent for MyD88, and expressing or not Ly49H (Fig 3). At d3 after MCMV infection, the Ly49H^+^ NK cells from BALB/c-Ly49H^+^ MyD88^-/-^ mice were clearly activated although to a significantly lesser extent than in BALB/c-Ly49H^+^ animals (Fig 3A–3D and S4A and S4C Fig). Diminished IL-12 and type I IFN production by DC as well as cell-intrinsic loss of IL-18 signaling could all have contributed to the decrease in NK cell proliferation, production of IFN-gamma and expression of Granzyme B observed in MyD88^-/-^ mice, according to previous reports which analyzed the contribution of these different responses during MCMV infection [23–26, 35, 36, 47, 48]. In any case, at d6 after MCMV infection, the activation of Ly49H^+^ NK cells was higher in BALB/c-Ly49H^+^ MyD88^-/-^ mice as compared to BALB/c-Ly49H^+^ animals (Fig 3A–3D). Moreover, NK cell depletion led to a significant increase in viral replication not only in BALB/c-Ly49H^+^ mice but also in BALB/c-Ly49H^+^ MyD88^-/-^ animals (Fig 3E and S4D Fig). Thus, NK cells can be sufficiently activated so as to contribute significantly to the control of MCMV infection despite a primary immune deficiency that abrogates viral sensing by DC through TLR7/9, as well as the responses to IL-18 and all other IL-1 family cytokines, provided that the NK cells can directly recognize virally infected cells through triggering of a dedicated NK cell activating receptor. Ly49H^+^ NK cells from BALB/c-Ly49H^+^ IFNAR^-/-^ mice were significantly activated at d3 and d6 post infection (Fig 3A–3D), to levels similar to those observed in BALB/c-Ly49H^+^ MyD88^-/-^ mice. However, in contrast to the situation in the latter animals, in BALB/c-Ly49H^+^ IFNAR^-/-^ mice, viral replication did not appear to be curtailed by NK cell activity, since it was already very high in untreated mice and was not increased further upon NK cell depletion (Fig 3E). These results suggest that Ly49H^+^ NK cells are similarly activated in BALB/c-Ly49H^+^ IFNAR^-/-^ and BALB/c-Ly49H^+^ MyD88^-/-^ mice but fail to control viral replication in the former because the absence of IFN-I response allows fast and widespread virus replication overwhelming the antiviral functions of Ly49H^+^ NK cell. Thus, MyD88 but not IFNAR deficiency can be compensated at least in part by direct sensing of infected cells by NK cells in our experimental model of systemic MCMV infection.

### NK cell activation in MyD88^-/-^ mice does not require cell-intrinsic Ly49H signals but likely relies on residual IL-12 responses

A strong activation of Ly49H^-^ NK cells was observed in all infected mice (Fig 3F–3I and S4A and S4B Fig). Hence, NK cell activation in MyD88^-/-^ mice did not require cell-intrinsic Ly49H signals. IL-12 and IL-18 production were decreased in MCMV-infected BALB/c MyD88^-/-^ and BALB/c-Ly49H^+^ MyD88^-/-^ mice (S4E Fig). However, similarly to what happened with the IFN-I response, IL-12/18 stimulated genes were strongly induced in infected MyD88^-/-^ mice (S4F and S4G Fig). Hence, persistence of NK cell activation in MyD88^-/-^ mice likely resulted from residual IL-12 production and subsequent IL-12-dependent responses.

### The combined deficiency for Ly49H and MyD88 prevents the expansion of antiviral CD8 T cells

**Fig 4:**
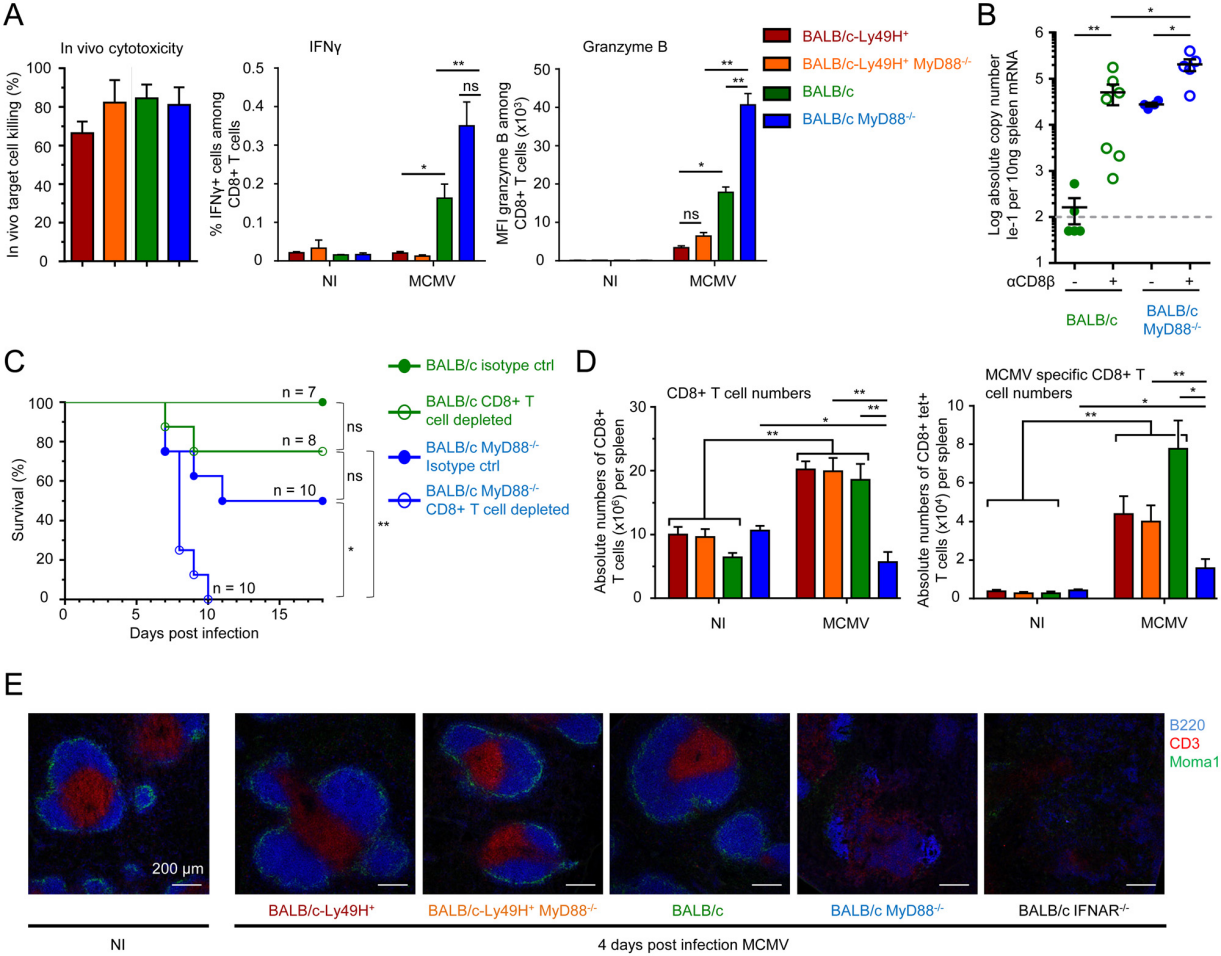
Impact of MyD88 or Ly49H deficiencies on antiviral CD8 T cell responses. (A-C) Analysis of antiviral CD8 T cell effector functions. (A) *In vivo* cytotoxicity, *ex vivo* IFN-γ production and Granzyme B expression by splenic CD8 T cells from BALB/c-Ly49H^+^, BALB/c-Ly49H^+^ MyD88^-/-^, BALB/c and BALB/c MyD88^-/-^ mice at d0 and 6 post infection. Data (mean ± SEM) are shown from 3 pooled independent experiments each with 2 or 3 mice per group. (B-C) Impact of CD8 T cell depletion on disease in BALB/c and BALB/c MyD88^-/-^ mice. Mice were depleted of CD8 T cells and infected with 2.5x10^3^ pfu MCMV (B) or with 8x10^3^ pfu MCMV (C). Splenic viral titers were measured at d6 post infection (B) or mortality was monitored daily (C). For B, data (mean±SEM) are represented from 2 pooled independent experiments each with 2 to 4 mice per group. For C, data show the percent survival from 2 pooled independent experiments each with 3 to 5 mice per group, n represents the total number of mice per group. (D) Enumeration of total (left panel) and anti-IE-1 (right panel) CD8 T cells in the spleens of d5 MCMV-infected mice. Results (mean±SEM) are shown from 4 pooled independent experiments each with 3 mice per group. (E) Immunohistological analysis of tissue damage to the spleen in d4 MCMV infected mice. Mice were infected with 10^4^ pfu MCMV. Spleen were harvested at d4 and stained to evaluate the integrity of the T cell zone as assessed by examining its marginal zone boundary (CD169) and its T cell (CD3) and B cell (B220) zones. Results are shown for one representative mouse per experimental group from 3 independent experiments each with 3 mice per group.

We next investigated the functions and numbers of antiviral CD8 T cells in the different mouse strains under study. Contrary to our expectations, the *in vivo* cytotoxic activity of anti-MCMV CD8 T cells was similar in the four mouse strains examined at d6 after infection (Fig 4A and S5A Fig). The CD8 T cells from Ly49H^-/-^MyD88^-/-^ mice even exhibited a significantly stronger expression of IFN-γ and Granzyme B directly *ex vivo* without any restimulation (Fig 4A and S5B Fig). Moreover, depletion of CD8 T cells led to a significant increase of viral replication both in BALB/c and BALB/c MyD88^-/-^ animals (Fig 4B and S5B Fig). Thus, during MCMV infection, CD8 T cells are hyperactivated in BALB/c MyD88^-/-^ animals and contribute to the control of viral replication. Hyperactivated CD8 T cell responses can contribute to liver immunopathology and death during MCMV infection [49]. However, CD8 T cell depletion led to a higher mortality of BALB/c MyD88^-/-^ mice (Fig 4C), ruling out a deleterious role of CD8 T cells in our experimental settings. Upon infection, there was a strong and significant increase in the numbers of total and anti-MCMV CD8 T cells in all of the three mouse strains expressing MyD88 and/or Ly49H, but not in BALB/c MyD88^-/-^animals (Fig 4D). Hence, since the efficiency of viral control *in vivo* by CD8 T cells depends on the local effector-to-target ratio in infected tissues [50], the enhanced susceptibility of BALB/c MyD88^-/-^ mice to MCMV infection likely resulted in part from their failure to expand their antiviral CD8 T cells. As compared to the other mouse strains, the BALB/c MyD88^-/-^ mice exhibited a significant increase in their splenic expression of genes associated with CD8 T cell exhaustion or with fibrosis and a decrease in the expression of the gene signature of red pulp macrophages (S5C and S5D Fig). This is consistent with the early and strong depletion of those cells previously reported in mice lacking efficient antiviral NK cell responses and infected with a high dose of MCMV [51]. At d4 post infection, BALB/c MyD88^-/-^ and BALB/c IFNAR^-/-^ mice also harbored a marked disruption of their spleen architecture (Fig 4E). Altogether, these observations suggest that early, strong and irreversible damage is caused to secondary lymphoid organs upon infection in Ly49H^-/-^MyD88^-/-^animals. This possibly is a consequence of persistently high levels of viral replication that cause a loss of the support function of these organs for all T cells and favor exhaustion of antiviral CD8 T cells. The early and profound loss of splenic red pulp macrophages and stromal cells occurring in BALB/c MyD88^-/-^animals, as witnessed by microarray analysis, likely contribute significantly to the loss of the specific micro-anatomical niches required for sustaining high numbers of antiviral CD8 T cells. Thus, either efficient antiviral NK cell activity or MyD88 functions are necessary to promote antiviral CD8 T cell responses while simultaneous failure of both responses strongly compromises antiviral adaptive immunity.

## Discussion

IFN-I responses are essential for defense against most viral infections in mice and men [2, 52–54]. However, it has been claimed that the requirements for pDC and endosomal TLR in IFN-I production differ fundamentally during natural infections of men and experimental challenges of laboratory mice [8], since IRAK4 or MyD88 genetic deficiencies only increase susceptibility to viruses in mice. However, whether IFN-I responses are impaired in infected MyD88^-/-^ mice and whether this contributes to their enhanced susceptibility to viral infections had not previously been rigorously examined. Moreover, it is unknown whether other immune responses could compensate for MyD88 deficiency during resistance to viral infections in mice as must occur in humans.

**Fig 5:**
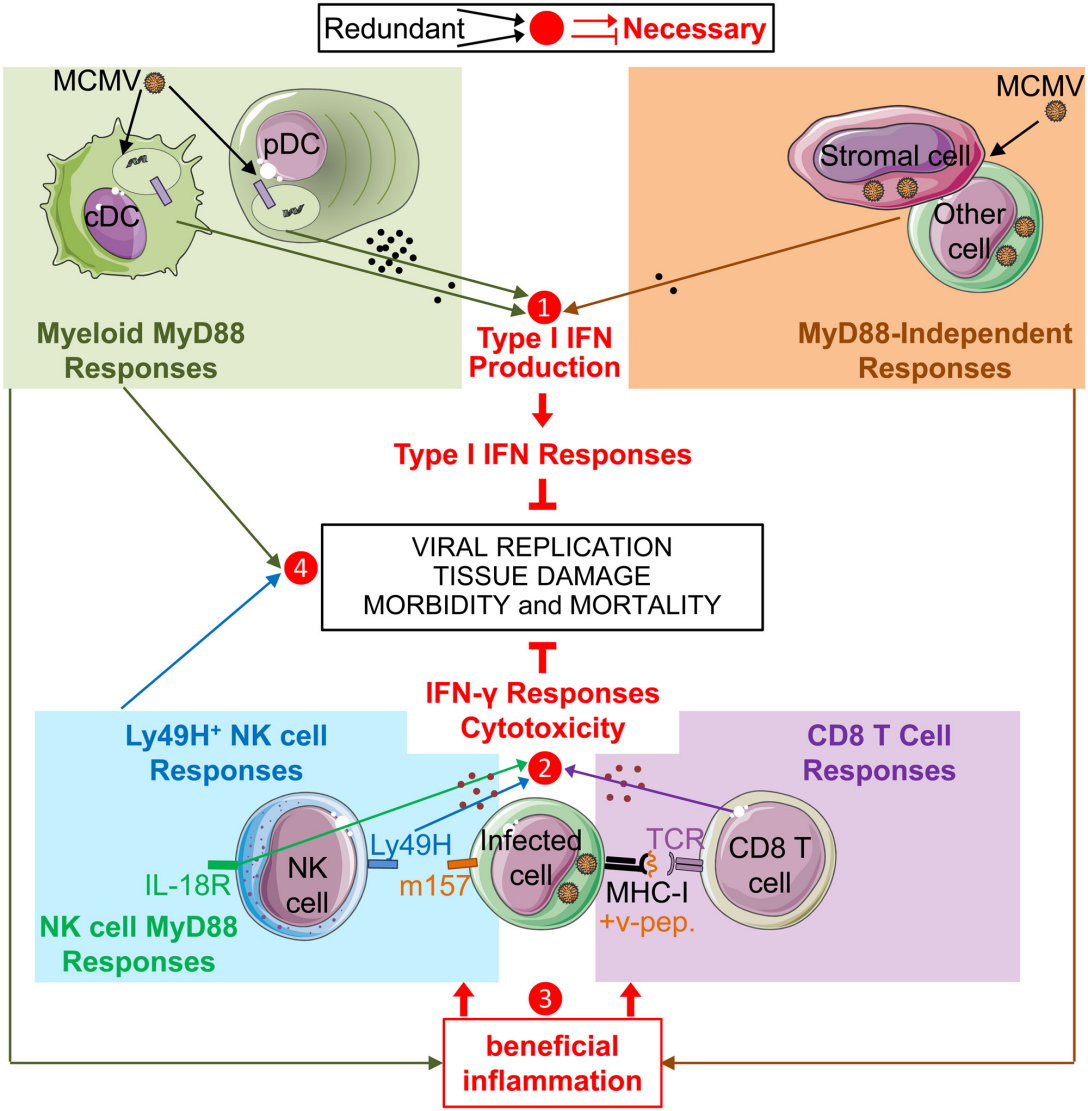
Model of redundancies and complementarities between molecular sensors and cell types for mounting the IFN-I, IFN-γ and cytotoxic cellular immune responses which are necessary for control of MCMV infection. Based on our own data as well as on many other studies published previously, we propose a model whereby IFN-I, IFN-γ and cytotoxic responses are all necessary for immune defenses against MCMV infection (red lines and text), but access to these functions can be promoted by a number of partly redundant and/or complementary pathways (arrows converging towards a number). IFN-I, IFN-γ and cytotoxic cellular immune responses are critical for control of viral replication, prevention of excessive tissue damage and overall resistance of the host in terms of morbidity and mortality. Several cell types and pathways can lead to IFN-I production. Early after intra-peritoneal injection, MCMV infects stromal cells and other cell types, inducing an IFN-I production by those cells independently of MyD88 and TLRs. In addition, viral particles or material derived from infected cells can be engulfed by cDC and pDC to promote their production of high levels of IFN-I or other cytokines upon triggering of the TLR7/9-to-MyD88 signaling cascade. In our experimental settings, MyD88/TLR9 responses of DC and MyD88-independent responses of infected cells are redundant for the induction of strong IFN-I responses, even though only very low to undetectable levels of IFN-I are produced in the absence of MyD88 responses (❶). In parallel, NK cells and CD8 T cells are able to produce IFN-γ and to specifically recognize and kill MCMV infected cells, via the activation receptor Ly49H or via their TCR, respectively. Cell-mediated immune control of viral replication can thus be performed by both NK and CD8 T cells which can largely compensate one another for this function (❷). However, the antiviral functions of NK and CD8 T cells are not strictly redundant but partly complementary, since, for example, the absence of CD8 T cell responses might increase the risk of selection of viral mutants able to escape NK cell control. MyD88 responses can promote the activation of NK and CD8 T cells via TLR-dependent activation of myeloid cells and/or through cell-intrinsic effects. However, MyD88 responses are not necessary for this function in mice expressing NK cell activation receptors able to directly sense MCMV-infected cells. In these mice, in addition to IFN-I production, MyD88-independent responses from infected cells might provide the other beneficial inflammatory signals necessary for this function (❸). Functional redundancies might also exist between MyD88 responses and NK cell activity for prevention of excessive damage to lymphoid tissues (❹), in order to preserve their micro-anatomical niches supporting the proliferation and survival of CD8 T cells.

Our results show that IFN-I responses are critical for defense against MCMV infection in mice. However, in our experimental settings, pDC and MyD88 are not required for the induction of protective IFN-I responses while they are crucial for high systemic production of these cytokines. Contrary to expectations [8, 11, 12], in mice, high systemic production of IFN-I resulting from endosomal sensing of viruses by pDC is dispensable for relatively efficient intrinsic, innate and adaptive immune responses to our model of systemic infection by a herpes virus. This observation is consistent with the redundancy of pDC and TLR7/9 functions but not IFN-I responses for antiviral defense in humans [2, 6–8, 53, 54]. Moreover, even if mice deficient for MyD88 are more susceptible than wild-type animals to MCMV infection, this can be compensated by other modalities of innate sensing of the infection, namely in our experimental set-up by direct NK cell recognition of infected cells. Hence, while IFN-I, IFN-γ and cytotoxic responses are all necessary for immune defenses against MCMV infection and most other experimental viral infections in mice, access to these functions can be promoted by a number of complementary and/or partly redundant pathways. These include the triggering of different innate immune recognition receptors and the mobilization of distinct cell types with overlapping antiviral activities (Fig 5). Indeed, Ly49H^+^ NK cells can promote early control of MCMV replication in the absence of CD8 T cells [55, 56] and *vice versa* [57, 58], likely because they exert partly overlapping functions including IFN-γ production and cytotoxicity [59–62] (Fig 5). However, in RAG-deficient C57BL/6 mice lacking B and T cell responses, immune escape variants mutated for m157 are selected over time and ultimately cause the death of the mice after several weeks [55, 56]. Hence, context-dependent redundancies in innate and adaptive antiviral immune responses likely contribute to the robustness of host defenses under physiological conditions despite the immune evasion strategies developed by many viruses, as illustrated also by the demonstration of redundancies and complementarities between NK cells, CD8 T cells and CD4 T cells in the prevention of MCMV reactivation in latently infected mice lacking humoral immunity [63]. It is likely that other innate immune effector cells contribute to context-dependent redundancies in antiviral defense mechanisms in mice, including NK T cells [64–68] or γδ T cells [69, 70]. Similarly, the absence of overtly increased susceptibility to viruses in patients genetically deficient for endosomal TLR activity due to loss-of-function mutations in *MYD88* or *IRKA4* likely results from their preserved ability to mount protective intrinsic and innate immune responses that efficiently control primary infections. These involve not only IFN-I responses but also innate cytotoxic responses by NK, NK T or γδ T cells [71–73], before adaptive immunity induction. However, it is possible that pDC and MyD88 are necessary for efficient host defense under specific conditions of viral infections, including during herpesvirus infections consecutive to bone marrow transplantation where adaptive immunity and some innate immune responses are suppressed and the conditions of viral replication may be analogous to a high dose challenge.

Our study demonstrates that even undetectably low levels of IFN-I/III are sufficient to induce a strong and widespread expression of genes involved in cell-intrinsic antiviral immunity during a systemic viral infection, including in MyD88^-/-^ mice. This observation is consistent with a recent report showing that strongly increasing IFN-I production by pDC during MCMV infection, while preserving other antiviral immune responses, does not improve control of viral replication [74]. On the contrary, high systemic IFN-I production by pDC might even delay the induction of antiviral adaptive immunity due to negative effects on the DC and CD8 T cell compartments [39, 75]. Hence, delivery of small amounts of IFN-I in a tightly controlled spatio-temporal manner could be a better way to treat certain viral infections or cancers than administration of high systemic doses of cytokines. The latter treatment may not induce better cell-intrinsic immunity but rather induce systemic responses associated with severe side effects [1]. In addition, given current technological limits to cytokine detection, our results suggest that to investigate whether a cytokine plays a physiological role in a disease, one should quantitate the effect of abrogating cytokine-mediated signaling on disease evolution, and functionally assess how perturbing cytokine activity affects disease outcome, rather than only titrating cytokine concentration.

One may wonder why infected hosts produce high systemic levels of IFN-I if very low to undetectable levels of these cytokines are sufficient to induce a strong expression of ISG and their downstream protective antiviral functions. One possibility is that high systemic production of IFN-I by spleen pDC in the absence of protective NK cell responses is necessary to promote defense or homeostasis of distant organs. It was recently published that elevated levels of IFN-I has systemic effects which promote epithelial turnover and wound repair [76]. However, since MyD88-deficient mice harbor only a mild increase in susceptibility to MCMV infection as compared to IFNAR-deficient mice, these types of systemic IFN-I responses might be rather required to prevent concomitant heterologous infections or for defense against viruses able to cause chronic infections, by allowing a systemic state of pathogen alert with induction of protective responses in all tissues. Indeed, a similar role was recently reported for IFN-γ production by tissue-resident memory CD8 T cells, whereby it induced tissue-wide responses able to protect the skin against viral variants able to escape adaptive immunity or against heterologous infections [77, 78].

In d6 infected BALB/c-Ly49H^+^ MyD88^-/-^ mice, not only Ly49H^+^ but also Ly49H^-^ NK cells are significantly more activated than in BALB/c-Ly49H^+^ mice. NK cells are also significantly activated in infected animals deficient in Ly49H and MyD88 signaling. Hence, NK cells do not require direct sensing of infected cells for their activation, even at late time points after infection. Rather Ly49H protects mice against MCMV by allowing cytokine-activated NK cells to specifically recognize and kill infected cells. The mechanisms promoting NK cell activation in MyD88^-/-^ mice merit further study. IL-12 and IL-18 play important roles in this process in wild type animals [35, 36, 47]. The analysis of the expression pattern of target genes of these cytokines in the spleen of infected mice showed that a significant and sustained induction of cytokine responses are still observed in MyD88^-/-^ animals despite a dramatic decrease in the expression of the cytokines themselves. Hence, similarly to IFN-I, IL-12 requires MyD88 for high level production but not for functional induction of NK cell activation.

Our result showed that NK cell sensing and killing of infected cells can compensate MyD88 but not IFNAR deficiency for the control of systemic MCMV infection in BALB/c mice. In contrast, others have reported that IFN-I responses are not required for NK cell activation and antiviral activity in C57BL/6 mice [42, 79]. This might be due in part to differences between C57BL/6 and BALB/c mice. Alternatively, this might be due to differences between virus strains. In the other studies, relatively low inoculum doses of *in vitro* produced viruses were used. Viruses deriving from the pSM3fr parental strain are highly attenuated *in vivo* due to a mutation in the viral MCK-2 chemokine [80]. Under these experimental settings, Ly49H^+^ NK cell responses were shown to completely or moderately compensate deficient IFN-I responses to promote control of viral replication. Further, NK cell activity was reported to be dispensable for late (d7) control of viral replication in C57BL/6 mice competent for IFN-I responses in contrast to our observations using BALB/c-Ly49H^+^ mice infected with moderate doses of salivary gland virus. The morbidity and mortality associated with the infections was not documented [42, 79]. Hence, it is possible that the previously reported compensation between IFNAR deficiency and NK cell activity resulted in part from the use of highly attenuated conditions of infection allowing clearance of MCMV and survival even in mice deficient for both IFNAR and Ly49H.

In our study, the higher susceptibility to MCMV of BALB/c MyD88^-/-^ mice as compared to BALB/c-Ly49H^+^ MyD88^-/-^ or BALB/c mice was associated with a dramatic decrease in the number of antiviral and total CD8 T cells in the spleen. BALB/c MyD88^-/-^ mice harbor a striking disruption of their spleen architecture as early as d4 after infection. Microarray analyses showed a strong induction of genes associated with fibrosis and a downregulation of the red pulp macrophage transcriptomic signature at d6 post-infection in the spleen of BALB/c MyD88^-/-^ animals. Hence, in the absence of efficient antiviral NK cell responses, MyD88 responses are required to protect the secondary lymphoid organs from extensive tissue damage. This includes preventing the loss of cell types and micro-anatomical structures necessary to support protective antiviral immune adaptive responses.

In summary, our study shows that MyD88 responses are dispensable for the induction of protective IFN-I responses against a systemic herpesvirus infection in mice. We show that the enhanced susceptibility of MyD88-deficient mice to MCMV infection is not attributable to loss of IFN-I responses, to decreased NK cell activation or to compromised CD8 T cell priming. Rather, it results from severe tissue damage to the spleen leading to a failure to sufficiently amplify the compartment of effector antiviral CD8 T cells. Moreover, we show that direct recognition and sensing of virus-infected cells by NK cells can compensate for MyD88- but not IFNAR-deficiency and promote resistance to a systemic MCMV infection in our experimental settings. These results challenge our current understanding of the requirements for the induction of protective responses to systemic viral infections in mice, in particular the role of pDC and endosomal TLR7/9 [8, 10–12, 24–27]. These results are consistent with the conclusions drawn from the studies of human patients suffering from primary immunodeficiencies [2, 6–8, 53, 54]. Hence, immune responses might not differ between mice and men as much as is sometimes suggested. Rather, it is important to define experimental settings in mice that best reflect clinical observations in humans, in order to take advantage of this versatile animal model to advance basic immunological knowledge in a way that might benefit human health [81]. Early disruption of lymphoid organ architecture during human immunodeficiency virus infection has been proposed as an important cause for the failure of the host to control viral replication and for disease development [82]. Understanding the mechanisms leading to the disruption of the architecture of lymphoid organs early during viral infections in mice and how MyD88 or NK cell responses can prevent this process could open novel avenues to treat viral infections in humans.

## Materials and Methods

### Ethics statement

The animal care and use protocols (ID no. 11-09/09/2011) were designed in accordance with national and international laws for laboratory animal welfare and experimentation (EEC Council Directive 2010/63/EU, September 2010), and approved by the Marseille Ethical Committee for Animal Experimentation (registered by the Comité National de Réflexion Ethique sur l’Expérimentation Animale under no. 14).

### Mice and *in vivo* treatments

8 to 12 week old mice of different strains were used for experiments (see supplemental materials and methods in S1 File). Animals were bred under pathogen-free conditions at CIML. Infections were initiated at d0 by i.p. injection of 2.5 x 10^3^ pfu salivary gland-extracted MCMV K181 v70 [23, 49] or Δm157 [45] strains (third and second *in vivo* passages, respectively), unless specified otherwise. pDC, NK cells or CD8 T cells were depleted *in vivo* by intraperitoneal delivery of 500μg anti-Bst2 (120G8) mAb, 100μg anti-NK1.1 mAb or 150μg anti-CD8β mAb. Antibodies were injected on d-1 before MCMV infection, followed by injections on d1, 3 and 5 for NK cell and pDC depletion, and on d2 and 7 for CD8 T cell depletion. Control mice were treated with Rat IgG. Cell depletion efficiency was assessed by flow cytometry as detailed in S1 File. Bst2 expression was increased on B cells, NK cells and cDC in infected mice. However, even under these conditions, the levels of Bst2 expressed by these cells were much lower than that observed on pDC. No depletion of B cells, T cells and NK cells was observed in our experimental conditions, although we cannot completely exclude that a very small fraction of some of these cell types was affected due to a higher expression of Bst2, for example plasmocytes. However, the numbers of cDC in infected mice treated with anti-Bst2 antibody was half that of infected animals treated with isotype control (S6 Fig).

### Spleen preparation and flow cytometry analysis

Spleen leukocyte suspensions were prepared using DNAse I and collagenase D [23], stained as detailed in S1 File, and acquired on a FACSCanto II Flow Cytometer (BD Bioscience).

### mRNA preparation, real-time PCR experiments, quantification of viral titers and microarray experiments

Pieces of spleen were harvested and stabilized overnight in RNAlater solution (Qiagen). High-quality total RNA was prepared and used for qRT-PCR or microarrays as previously described [23]. Relative gene expression was calculated with the ΔΔCt method using *Hprt* as housekeeping gene for normalization. Viral titers were measured as absolute levels of expression of the *Ie1* gene [23]. Microarray analyses were performed as previously described [23]. Gene set compositions are given in S1 Table. Data have been deposited in the GEO database under reference GSE62729.

### Quantification of serum IFN-α levels

Serum IFN-α2/α4 levels were determined by ELISA (eBioscience) according to the manufacturer’s instructions.

### *In vivo* cytotoxicity assay

Antigen-specific CD8 T cell–mediated *in vivo* cytotoxicity was assayed as described [83].

### Enumeration of, and effector molecule expression by, antiviral CD8 T cells

Splenic lymphocytes from mice infected with MCMV were isolated at d6 post infection and CD8 T cells were analyzed for H-2L(d)/IE-1(168YPHFMPTNL176) binding and intracellular IFN-γ or Granzyme B expression.

### Morbidity and mortality

Infected mice were monitored daily for signs of morbidity (weight loss, piloerection, hunched posture and lethargy). Imminent death was defined as loss of 20% initial body weight or development of severe lethargy (unresponsiveness to touch) established in a preliminary experiment using death as the endpoint.

### Histology

Spleen section were prepared, stained and imaged as previously described [28] and detailed in S1 File.

### Statistical analyses

Statistical analyses were performed using a nonparametric Mann-Whitney test performed with Prism 6 (GraphPad Software) for all experiments except survival analyses where a Mantel-Cox test was used. ns, non significant (p > 0.05); *p < 0.05; **p < 0.01; ***p < 10^–3^.

## Supporting Information

S1 Fig Mortality and viral loads in BALB/c and BALB/c-Ly49H^+^ mice infected with a Δm157 strain of K181 MCMV. Mice were infected with 2.2x10^4^ pfu of salivary gland-extracted Δm157 K181 MCMV. (A) This inoculum dose was chosen as close to the LD_50_ for BALB/c mice, as determined upon *in vivo* titration of the virus in this mouse strain. The viral inoculums used for the titration are indicated on the survival curve. Data are from 1 experiment with 5 mice per group. (B) Mortality was monitored daily; n represents the number of mice per group. (C) Splenic viral loads at d3 post infection are shown. Dashed line represents the limit of detection. Data (mean±SEM) are represented from 1 experiment with 4 or 5 mice per group.
(PDF)

S2 Fig Absence of IFN-I production by pDCs does not strongly compromise IFN-I responses and MCMV control. (A) Verification of pDC depletion efficiency and analysis of IFN-β expression in pDCs. Data are shown for one representative mouse for each experimental group. Three independent experiments each with 3 mice per group were performed. Splenic pDCs were gated as CD3ε^-^CD19^-^NKp46^-^CD11c^int^SiglecH^+^ cells. (Top) The frequency of pDCs is shown in an uninfected and untreated animal, and at d1.5 after infection in one infected control mouse treated with rat IgG versus in one animal depleted of pDCs by *in vivo* administration of α120G8 antibodies. (Bottom) The frequency of pDCs expressing IFN-β directly *ex vivo* without any re-stimulation is shown in an uninfected animal, and at d1.5 after infection in one infected animal for each of the four mouse strains studied, BALB/c-Ly49H^+^, BALB/c-Ly49H^+^ MyD88^-/-^, BALB/c and BALB/c MyD88^-/-^ mice. (B) Impact of pDC depletion on splenic viral loads at d6 post infection in BALB/c mice. Dashed line represents the limit of detection. Data (mean±SEM) are represented from 2 pooled independent experiments each with 3 mice per group. (C) Splenic viral loads at d1.5 post infection with 2.5x10^3^ pfu MCMV in BALB/c and BALB/c TLR9^-/-^ mice. Dashed line represents the limit of detection. Data (mean±SEM) are represented from 1 experiment. (D) Frequency of IFN-β^+^ cells within splenic pDCs of BALB/c-Ly49H^+^, BALB/c-Ly49H^+^ MyD88^-/-^, BALB/c, BALB/c MyD88^-/-^ and BALB/c TLR9^-/-^ mice at d0 and d1.5 post infection. Results (mean±SEM) are represented from one experiment representative of two independent ones, each with 3 mice per group. (E-F) Gene set enrichment analysis (GSEA) results for examining enrichment of ISG expression in pairwise comparisons between d0 and d1.5 after infection in BALB/c-Ly49H^+^, BALB/c-Ly49H^+^ MyD88^-/-^, BALB/c and BALB/c MyD88^-/-^ mice. (E) Examples of raw GSEA results classically represented as enrichment plots. Each bar under the curves corresponds to the projection of one of the 1,648 ISG ProbeSets on the red-to-blue gradient representing all the 35,556 ProbeSets from the gene chip ranked from high expression at d1.5 to high expression at day 0. The more the GeneSet is differentially expressed between conditions, the more the bar code is shifted to one extremity. This is measured by two parameters. The normalized enrichment score (NES) represents the number and differential expression intensity of the genes enriched. The false discovery rate (FDR) statistical value (q) represents the likelihood that the enrichment of the GeneSet represents a false-positive finding (e.g., if q = 0.25, a similar enrichment is found in 25% of the random GeneSets used as controls). The absolute NES values vary between 1 (no enrichment) and 5 (maximal enrichment possible). The enrichment is considered significant for absolute NES values > 1 with an associated q value < 0.25. The result from each enrichment plot can be synthesized as a dot, bigger and darker for stronger and more significant enrichment, in a color matching that of the condition in which the GeneSet was enriched (blue for uninfected mice and red for infected mice). (F) Summary of GSEA results for all mouse strains and all time points after infection examined. (G) The heatmap shows the relative expression value for 100 ISG. Results shown are from the same 2 pooled independent experiments than in Fig 1D and 1E, each with 1 to 3 mice per group.
(PDF)

S3 Fig Impact of pDC depletion on mortality upon MCMV infection. BALB/c mice were treated by intraperitoneal delivery of 500μg α120G8 antibody or isotype control. Antibodies were injected on d-1 before MCMV infection, followed by injections every 2 days. Mice were infected with 2x10^4^ pfu MCMV. Mortality was monitored daily. Data show the percent survival from 1 experiment; n represents the number of mice per group.
(PDF)

S4 Fig Impact of MyD88 deficiency on NK cell and IL-12 responses during MCMV infection. (A-C) Analysis of IFN-γ, Ki67 and Granzyme B expression in NK cells at d6 post infection. Data are shown for one representative mouse for each experimental group. Three independent experiments each with 3 mice per group were performed. (A) Splenic NK were gated as TCRβ^-^CD19^-^NKp46^+^ cells, and split into Ly49H^+^ versus Ly49H^-^ subsets in Ly49H-expressing mouse strains. (B-C) IFN-γ versus Ki67 (dot plots) and Granzyme B (histograms) expression at d6 post infection in Ly49H^-^ (B) and Ly49H^+^ (C) NK cell subsets are shown for each mouse strain. (D) Verification of the efficiency of NK cell depletion. The frequency of NK cells is shown for one representative animal for each experimental group: in an uninfected and untreated animal, and at d6 after infection in one infected control mouse treated with rat IgG versus in one animal depleted of NK cells by *in vivo* administration of αNK1.1 antibodies. Three independent experiments each with 2 to 3 mice per group were performed. (E) Splenic expression of the *Il12b* and *Il18* genes at different time points after infection with 2.5x10^3^ pfu MCMV in BALB/c-Ly49H^+^, BALB/c-Ly49H^+^ MyD88^-/-^, BALB/c and BALB/c MyD88^-/-^ mice, as measured in microarrays. (F) GSEA results for examining enrichment of the expression of IL-12/IL-18 stimulated genes (n = 631) in pairwise comparisons between uninfected and infected animals at different time points after MCMV inoculation, in BALB/c-Ly49H^+^, BALB/c-Ly49H^+^ MyD88^-/-^, BALB/c and BALB/c MyD88^-/-^ mice. The results are shown as described for S2E–S2F Fig. (G) Heatmap showing the relative expression of selected IL-12/IL-18 stimulated genes. For (E-G), the results shown are from the same microarray data as used in Fig 1D and 1E.
(PDF)

S5 Fig Impact of MyD88 and Ly49H deficiencies on antiviral CD8 T cell responses and spleen damages. (A) *In vivo* cytotoxic activity of antiviral CD8 T cells at d6 post-infection in BALB/c-Ly49H^+^, BALB/c-Ly49H^+^ MyD88^-/-^, BALB/c and BALB/c MyD88^-/-^ mice. Unpulsed and IE-1 peptide-pulsed syngeneic splenocytes were stained with PKH26 and high versus low concentrations of CFSE. These target cells were then transferred into mice at d6 post-MCMV infection. The spleens were harvested 4h later to measure with PKH26^+^ transferred cells the relative frequency between unpulsed (CSFE^high^) versus pulsed (CSFE^low^) targets in each mouse and hence calculate the percent of specific *in vivo* killing by antiviral CD8 T cells. Data are shown for one representative mouse for each experimental group. Three independent experiments each with 2 or 3 mice per group were performed. (B) Verification of the efficiency of CD8 T cell depletion and analysis of IFN-γ and Granzyme B expression in CD8 T cells. Data are shown for one representative mouse for each experimental group. Two independent experiments each with 2 to 4 mice per group were performed. Splenic CD8 T cells were gated as NKp46^-^TCRβ^+^CD4^-^CD8α^+^ cells. In the upper row, the frequency of CD8 T cells is shown in an uninfected and untreated animal, and at d6 after infection in one infected control mouse treated with rat IgG versus in one animal depleted of CD8 T cells by *in vivo* administration of αCD8β antibodies. In the lower row, the frequency of CD8 T cells expressing IFN-γ directly *ex vivo* without any re-stimulation is shown in an uninfected animal, and at d6 after infection in one infected animal for each of the four mouse strains studied, BALB/c-Ly49H^+^, BALB/c-Ly49H^+^ MyD88^-/-^, BALB/c and BALB/c MyD88^-/-^ mice. Granzyme B expression on CD8 T cells is shown as histograms. (C) GSEA results for examining enrichment of the expression of genes associated to “exhausted T cells” (n = 86) or “RPM” (n = 106), or of genes co-regulated with COL6A1 (n = 95) or MMp19 (n = 98) in pairwise comparisons between Ly49H^-/-^MyD88^-/-^ mice and each of the 3 other mouse strains at d6 post infection. The results are shown as described for S2E and S2F Fig. (D) Heatmaps showing the relative expression of selected genes from the gene sets of panel C. For (C-D), the results shown are from the same microarray data as used in Fig 1D and 1E.
(PDF)

S6 Fig Anti-Bst2 antibody treatment promotes a selective but not entirely specific pDC depletion after MCMV infection. (A) Splenocytes from BALB/c mice were stained and analyzed by FACS at d0 or at d1.5 after infection with 2.5×10^3^ pfu MCMV. Different cell populations were selected (dot plots) and their Bst2 expression assessed by staining with 120G8 mAb (histograms). Results are shown from one representative mouse of 3 for each condition. (B) Control (Rat IgG) or pDC-depleted (120G8 mAb treated) BALB/c mice were infected or not with 2.5 × 10^3^ pfu MCMV. Splenocytes were stained and analyzed by FACS to assess the efficiency and selectivity of the depletion. T+B (CD3ε^+^CD19^+^), NK (CD3ε^-^CD19^-^NKp46^+^), cDC (CD3ε^-^CD19^-^NKp46^-^CD11c^high^), pDC (CD3ε^-^CD19^-^NKp46^-^CD11c^int^SiglecH^+^) and other cells («non NK-T-B-DC», CD3ε^-^CD19^-^CD11c^-^) were gated and their relative proportion indicated on the dot plots. The absolute numbers of splenocytes did not differ between the 2 groups of infected mice. Results are shown from one experiment representative of 3 independent ones, each with 2 to 3 mice per group.
(PDF)

S1 Table GeneSets used in bioinformatics analyses of microarray data. (XLSX)

S1 File Supplemental Materials and Methods. (DOCX)
