# Supplementary figures and images for: Natural Killer Cell Sensing of Infected Cells Compensates for MyD88 Deficiency but Not IFN-I Activity in Resistance to Mouse Cytomegalovirus
Source: PLoS Pathog. 2015 May 8;11(5):e1004897. doi: 10.1371/journal.ppat.1004897 (PMC4425567; doi:10.1371/journal.ppat.1004897)

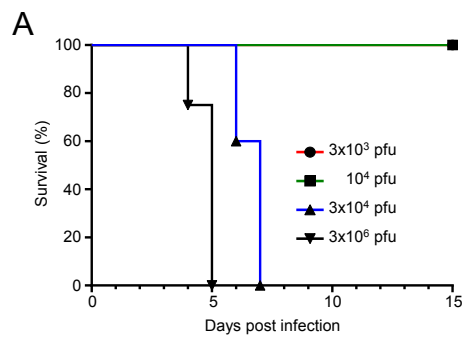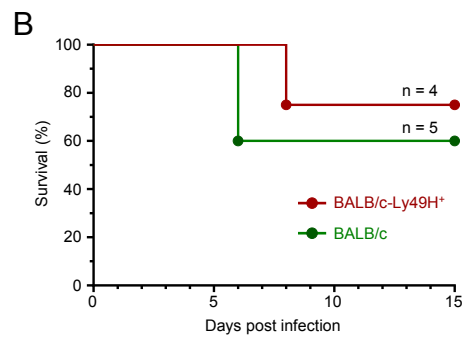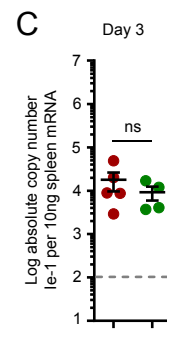

Supplement: S1 Fig — Mice were infected with 2.2x10^4^ pfu of salivary gland-extracted Δm157 K181 MCMV. (A) This inoculum dose was chosen as close to the LD50 for BALB/c mice, as determined upon in vivo titration of the virus in this mouse strain. The viral inoculums used for the titration are indicated on the survival curve. Data are from 1 experiment with 5 mice per group. (B) Mortality was monitored daily; n represents the number of mice per group. (C) Splenic viral loads at d3 post infection are shown. Dashed line represents the limit of detection. Data (mean±SEM) are represented from 1 experiment with 4 or 5 mice per group. (PDF) [file ppat.1004897.s001.pdf]

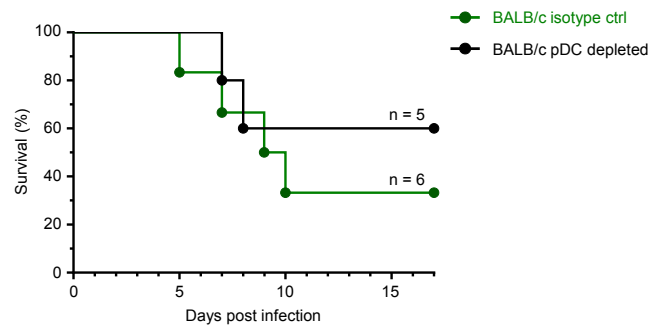

Supplement: S3 Fig — BALB/c mice were treated by intraperitoneal delivery of 500μg α120G8 antibody or isotype control. Antibodies were injected on d-1 before MCMV infection, followed by injections every 2 days. Mice were infected with 2x10^4^ pfu MCMV. Mortality was monitored daily. Data show the percent survival from 1 experiment; n represents the number of mice per group. (PDF) [file ppat.1004897.s003.pdf]

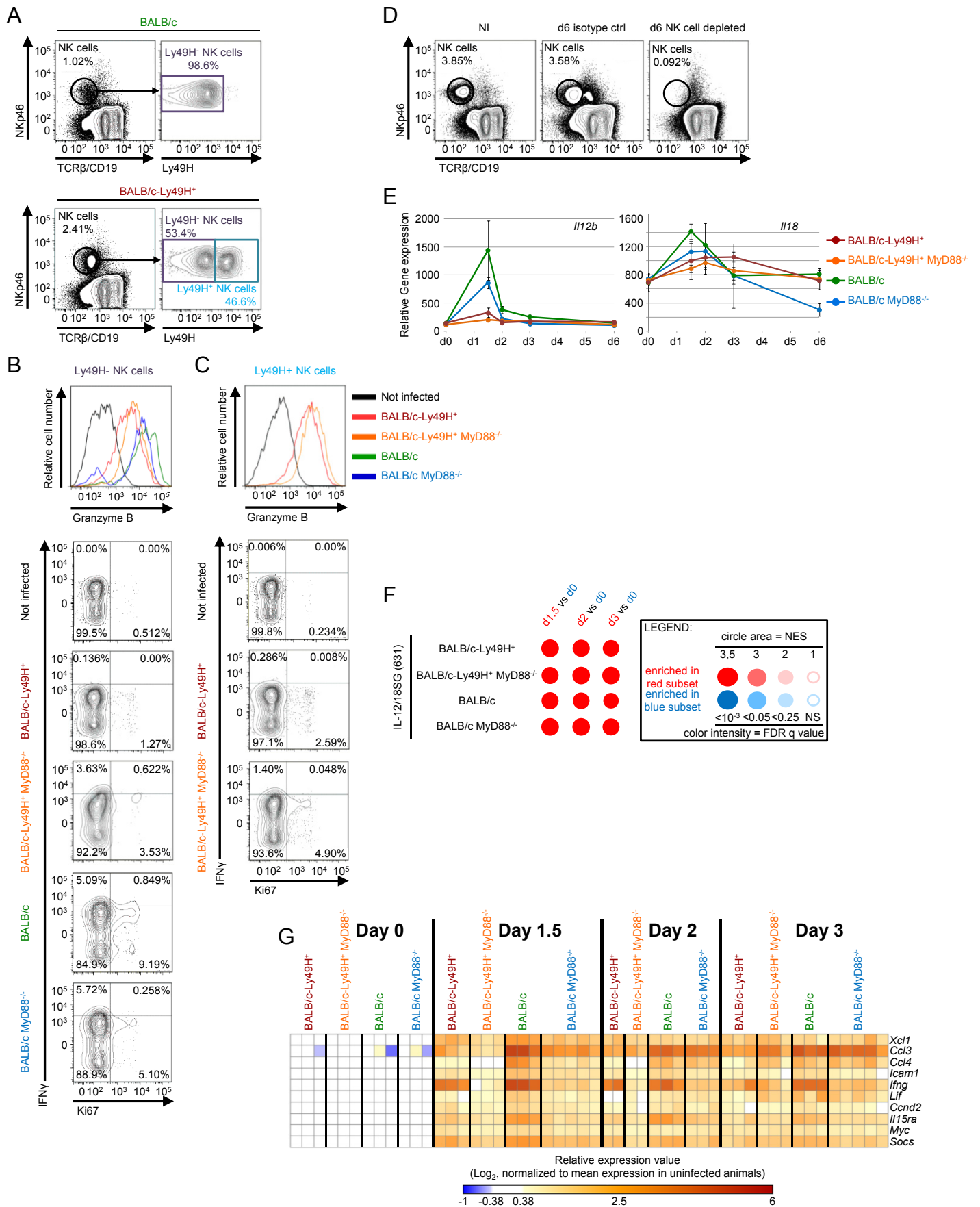

Supplement: S4 Fig — (A-C) Analysis of IFN-γ, Ki67 and Granzyme B expression in NK cells at d6 post infection. Data are shown for one representative mouse for each experimental group. Three independent experiments each with 3 mice per group were performed. (A) Splenic NK were gated as TCRβ-CD19-NKp46+ cells, and split into Ly49H+ versus Ly49H- subsets in Ly49H-expressing mouse strains. (B-C) IFN-γ versus Ki67 (dot plots) and Granzyme B (histograms) expression at d6 post infection in Ly49H- (B) and Ly49H+ (C) NK cell subsets are shown for each mouse strain. (D) Verification of the efficiency of NK cell depletion. The frequency of NK cells is shown for one representative animal for each experimental group: in an uninfected and untreated animal, and at d6 after infection in one infected control mouse treated with rat IgG versus in one animal depleted of NK cells by in vivo administration of αNK1.1 antibodies. Three independent experiments each with 2 to 3 mice per group were performed. (E) Splenic expression of the Il12b and Il18 genes at different time points after infection with 2.5x10^3^ pfu MCMV in BALB/c-Ly49H+, BALB/c-Ly49H+ MyD88-/-, BALB/c and BALB/c MyD88-/- mice, as measured in microarrays. (F) GSEA results for examining enrichment of the expression of IL-12/IL-18 stimulated genes (n = 631) in pairwise comparisons between uninfected and infected animals at different time points after MCMV inoculation, in BALB/c-Ly49H+, BALB/c-Ly49H+ MyD88-/-, BALB/c and BALB/c MyD88-/- mice. The results are shown as described for S2E–S2F Fig. (G) Heatmap showing the relative expression of selected IL-12/IL-18 stimulated genes. For (E-G), the results shown are from the same microarray data as used in Fig 1D and 1E. (PDF) [file ppat.1004897.s004.pdf]

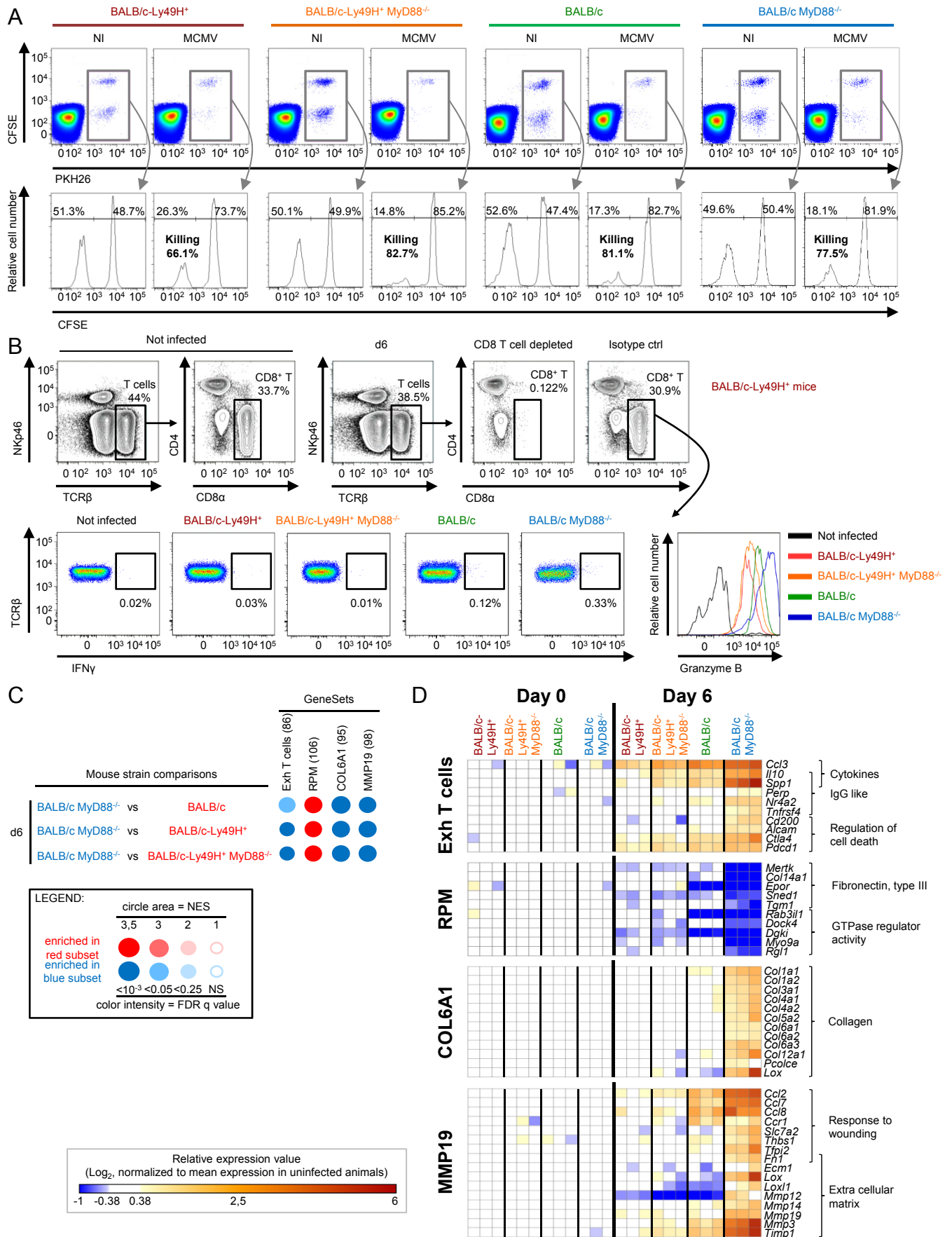

Supplement: S5 Fig — (A) In vivo cytotoxic activity of antiviral CD8 T cells at d6 post-infection in BALB/c-Ly49H+, BALB/c-Ly49H+ MyD88-/-, BALB/c and BALB/c MyD88-/- mice. Unpulsed and IE-1 peptide-pulsed syngeneic splenocytes were stained with PKH26 and high versus low concentrations of CFSE. These target cells were then transferred into mice at d6 post-MCMV infection. The spleens were harvested 4h later to measure with PKH26+ transferred cells the relative frequency between unpulsed (CSFEhigh) versus pulsed (CSFElow) targets in each mouse and hence calculate the percent of specific in vivo killing by antiviral CD8 T cells. Data are shown for one representative mouse for each experimental group. Three independent experiments each with 2 or 3 mice per group were performed. (B) Verification of the efficiency of CD8 T cell depletion and analysis of IFN-γ and Granzyme B expression in CD8 T cells. Data are shown for one representative mouse for each experimental group. Two independent experiments each with 2 to 4 mice per group were performed. Splenic CD8 T cells were gated as NKp46-TCRβ+CD4-CD8α+ cells. In the upper row, the frequency of CD8 T cells is shown in an uninfected and untreated animal, and at d6 after infection in one infected control mouse treated with rat IgG versus in one animal depleted of CD8 T cells by in vivo administration of αCD8β antibodies. In the lower row, the frequency of CD8 T cells expressing IFN-γ directly ex vivo without any re-stimulation is shown in an uninfected animal, and at d6 after infection in one infected animal for each of the four mouse strains studied, BALB/c-Ly49H+, BALB/c-Ly49H+ MyD88-/-, BALB/c and BALB/c MyD88-/- mice. Granzyme B expression on CD8 T cells is shown as histograms. (C) GSEA results for examining enrichment of the expression of genes associated to “exhausted T cells” (n = 86) or “RPM” (n = 106), or of genes co-regulated with COL6A1 (n = 95) or MMp19 (n = 98) in pairwise comparisons between Ly49H-/-MyD88-/- mice and each of the 3 ot [file ppat.1004897.s005.pdf]

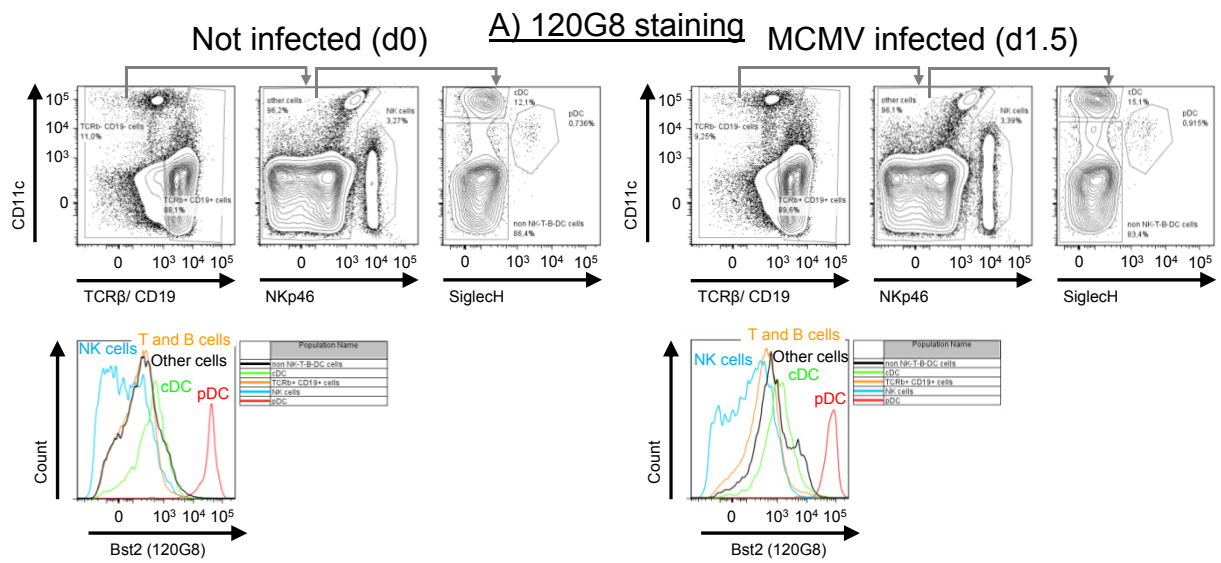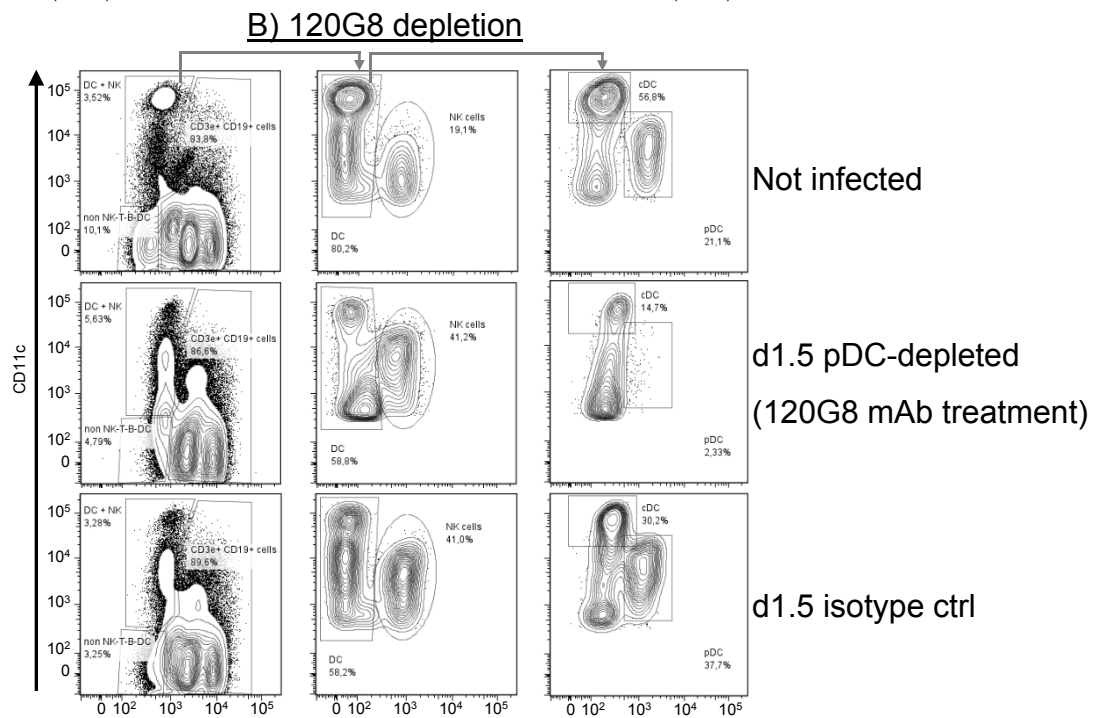

Supplement: S6 Fig — (A) Splenocytes from BALB/c mice were stained and analyzed by FACS at d0 or at d1.5 after infection with 2.5×10^3^ pfu MCMV. Different cell populations were selected (dot plots) and their Bst2 expression assessed by staining with 120G8 mAb (histograms). Results are shown from one representative mouse of 3 for each condition. (B) Control (Rat IgG) or pDC-depleted (120G8 mAb treated) BALB/c mice were infected or not with 2.5 × 10^3^ pfu MCMV. Splenocytes were stained and analyzed by FACS to assess the efficiency and selectivity of the depletion. T+B (CD3ε+CD19+), NK (CD3ε-CD19-NKp46+), cDC (CD3ε-CD19-NKp46-CD11chigh), pDC (CD3ε-CD19-NKp46-CD11cintSiglecH+) and other cells («non NK-T-B-DC», CD3ε-CD19-CD11c-) were gated and their relative proportion indicated on the dot plots. The absolute numbers of splenocytes did not differ between the 2 groups of infected mice. Results are shown from one experiment representative of 3 independent ones, each with 2 to 3 mice per group. (PDF) [file ppat.1004897.s006.pdf]
