# Supplementary material for: Natural Killer Cell Sensing of Infected Cells Compensates for MyD88 Deficiency but Not IFN-I Activity in Resistance to Mouse Cytomegalovirus
Source: PLoS Pathog. 2015 May 8;11(5):e1004897. doi: 10.1371/journal.ppat.1004897 (PMC4425567; doi:10.1371/journal.ppat.1004897)
Supplement: S2 Fig — (A) Verification of pDC depletion efficiency and analysis of IFN-β expression in pDCs. Data are shown for one representative mouse for each experimental group. Three independent experiments each with 3 mice per group were performed. Splenic pDCs were gated as CD3ε-CD19-NKp46-CD11cintSiglecH+ cells. (Top) The frequency of pDCs is shown in an uninfected and untreated animal, and at d1.5 after infection in one infected control mouse treated with rat IgG versus in one animal depleted of pDCs by in vivo administration of α120G8 antibodies. (Bottom) The frequency of pDCs expressing IFN-β directly ex vivo without any re-stimulation is shown in an uninfected animal, and at d1.5 after infection in one infected animal for each of the four mouse strains studied, BALB/c-Ly49H+, BALB/c-Ly49H+ MyD88-/-, BALB/c and BALB/c MyD88-/- mice. (B) Impact of pDC depletion on splenic viral loads at d6 post infection in BALB/c mice. Dashed line represents the limit of detection. Data (mean±SEM) are represented from 2 pooled independent experiments each with 3 mice per group. (C) Splenic viral loads at d1.5 post infection with 2.5x10^3^ pfu MCMV in BALB/c and BALB/c TLR9-/- mice. Dashed line represents the limit of detection. Data (mean±SEM) are represented from 1 experiment. (D) Frequency of IFN-β+ cells within splenic pDCs of BALB/c-Ly49H+, BALB/c-Ly49H+ MyD88-/-, BALB/c, BALB/c MyD88-/- and BALB/c TLR9-/- mice at d0 and d1.5 post infection. Results (mean±SEM) are represented from one experiment representative of two independent ones, each with 3 mice per group. (E-F) Gene set enrichment analysis (GSEA) results for examining enrichment of ISG expression in pairwise comparisons between d0 and d1.5 after infection in BALB/c-Ly49H+, BALB/c-Ly49H+ MyD88-/-, BALB/c and BALB/c MyD88-/- mice. (E) Examples of raw GSEA results classically represented as enrichment plots. Each bar under the curves corresponds to the projection of one of the 1,648 ISG ProbeSets on the red-to-blue gradient representing [file ppat.1004897.s002.pdf]

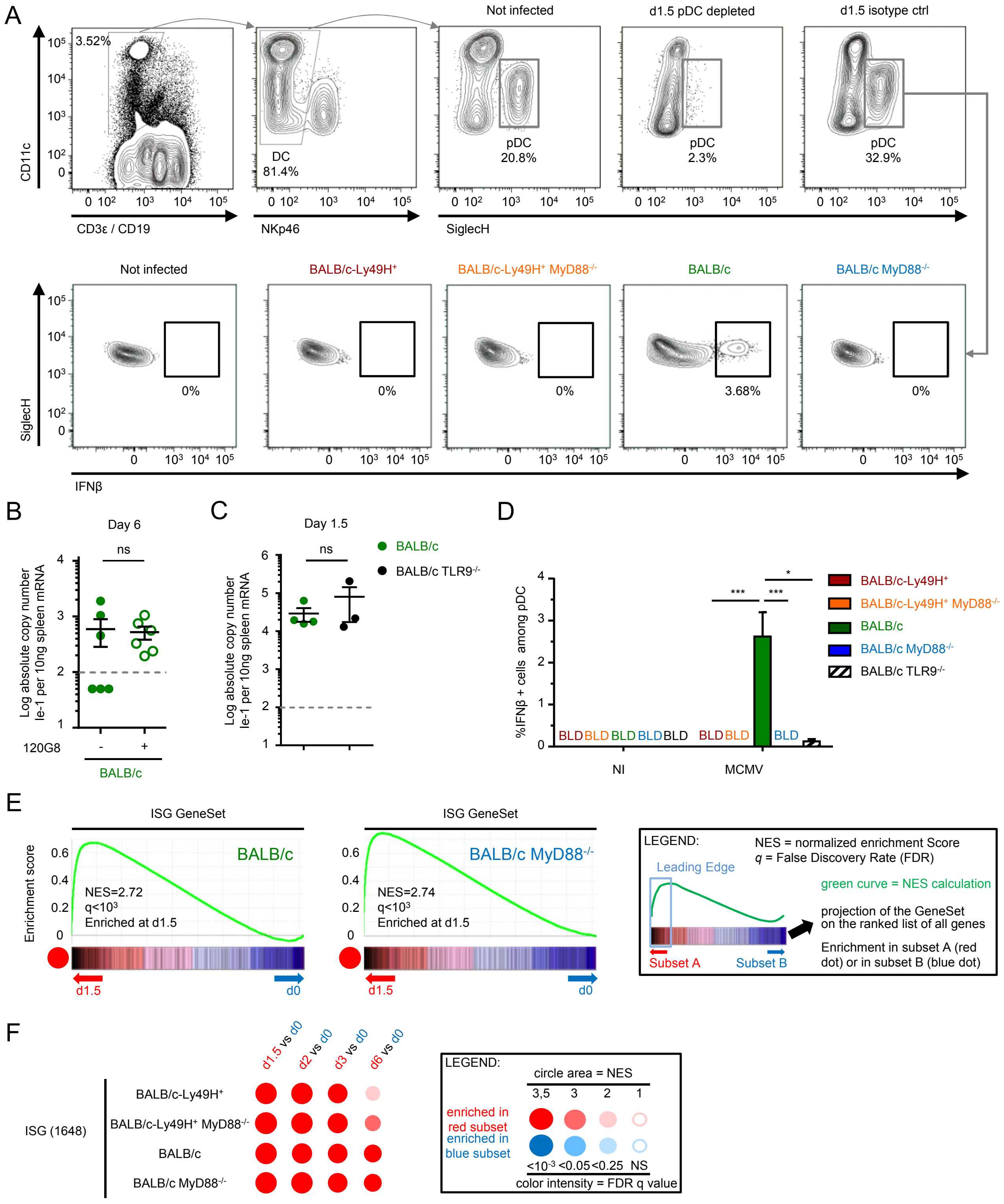
A
CD11c
CD3ε / CD19
3.52%
NKp46
DC
81.4%
SiglecH
Not infected
pDC
20.8%
d1.5 pDC depleted
pDC
2.3%
d1.5 isotype ctrl
pDC
32.9%
Not infected
BALB/c-Ly49H+
BALB/c-Ly49H+ MyD88-/-
BALB/c
BALB/c MyD88-/-
0%
0%
0%
3.68%
0%
IFNβ
B
Day 6
ns
Log absolute copy number
Ie-1 per 10ng spleen mRNA
120G8
BALB/c
C
Day 1.5
ns
BALB/c
BALB/c TLR9-/-
D
%IFNβ + cells among pDC
BLD BLD BLD BLD BLD BLD BLD BLD
NI
MCMV
***
***
*
BALB/c-Ly49H+
BALB/c-Ly49H+ MyD88-/-
BALB/c
BALB/c MyD88-/-
BALB/c TLR9-/-
E
ISG GeneSet
Enrichment score
BALB/c
NES=2.72
q<10³
Enriched at d1.5
d1.5
d0
ISG GeneSet
BALB/c MyD88-/-
NES=2.74
q<10³
Enriched at d1.5
d1.5
d0
LEGEND:
NES = normalized enrichment Score
q = False Discovery Rate (FDR)
Leading Edge
green curve = NES calculation
projection of the GeneSet on the ranked list of all genes
Enrichment in subset A (red dot) or in subset B (blue dot)
Subset A
Subset B
F
d1.5 vs d0
d2 vs d0
d3 vs d0
d6 vs d0
ISG (1648)
BALB/c-Ly49H+
BALB/c-Ly49H+ MyD88-/-
BALB/c
BALB/c MyD88-/-
LEGEND:
circle area = NES
3,5 3 2 1
enriched in red subset
enriched in blue subset
<10-3 <0.05 <0.25 NS
color intensity = FDR q value

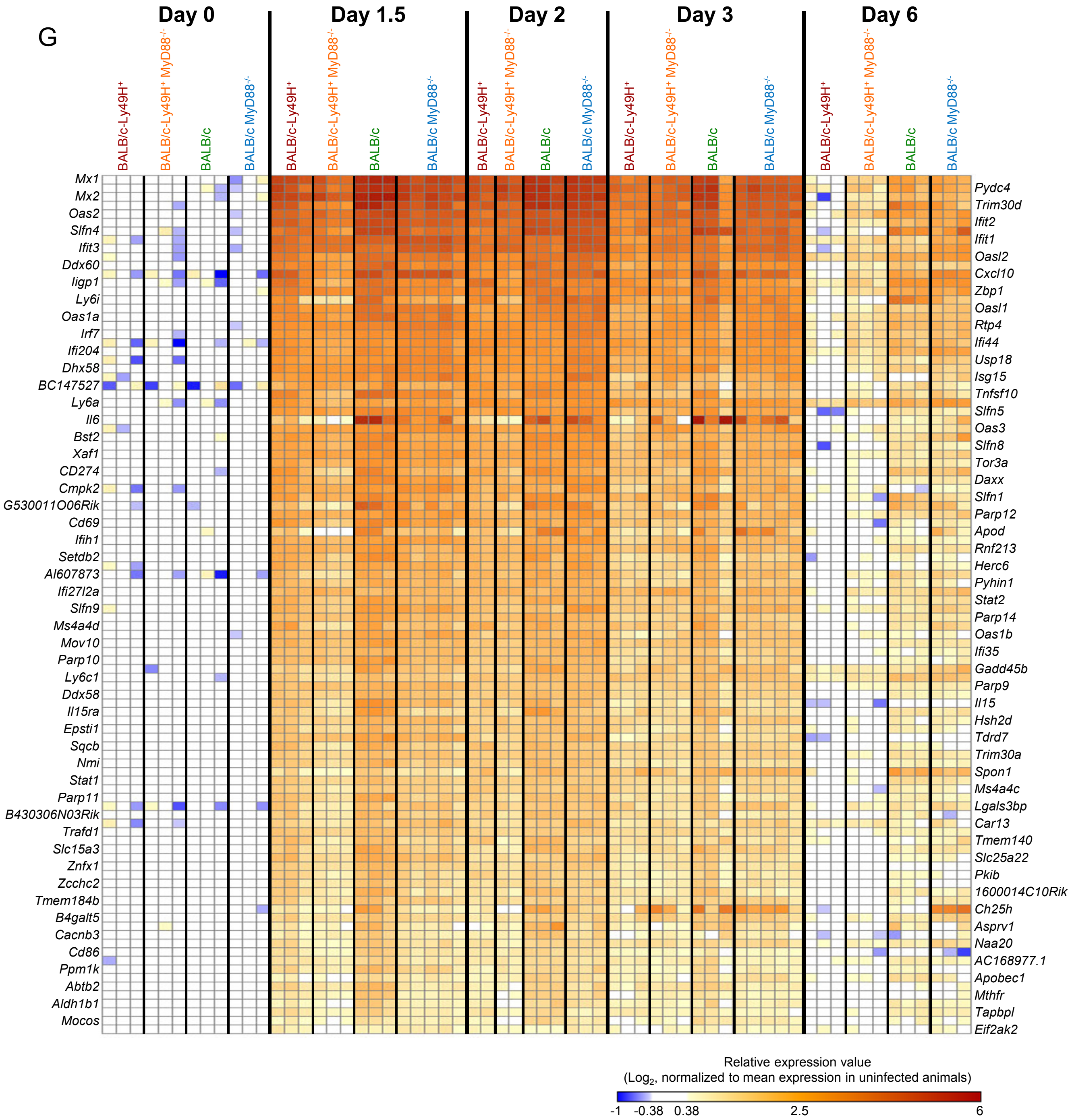
G
Day 0
Day 1.5
Day 2
Day 3
Day 6
BALB/c-Ly49H+
BALB/c-Ly49H+ MyD88-/-
BALB/c
BALB/c MyD88-/-
Mx1
Mx2
Oas2
Slfn4
Ifit3
Ddx60
Iigp1
Ly6i
Oas1a
Irf7
Ifi204
Dhx58
BC147527
Ly6a
Il6
Bst2
Xaf1
CD274
Cmpk2
G530011O06Rik
Cd69
Ifih1
Setdb2
AI607873
Ifi27l2a
Slfn9
Ms4a4d
Mov10
Parp10
Ly6c1
Ddx58
Il15ra
Epsti1
Sqcb
Nmi
Stat1
Parp11
B430306N03Rik
Trafd1
Slc15a3
Znfx1
Zcchc2
Tmem184b
B4galt5
Cacnb3
Cd86
Ppm1k
Abtb2
Aldh1b1
Mocos
Pydc4
Trim30d
Ifit2
Ifit1
Oasl2
Cxcl10
Zbp1
Oasl1
Rtp4
Ifi44
Usp18
Isg15
Tnfsf10
Slfn5
Oas3
Slfn8
Tor3a
Daxx
Slfn1
Parp12
Apod
Rnf213
Herc6
Pyhin1
Stat2
Parp14
Oas1b
Ifi35
Gadd45b
Parp9
Il15
Hsh2d
Tdrd7
Trim30a
Spon1
Ms4a4c
Lgals3bp
Car13
Tmem140
Slc25a22
Pkib
1600014C10Rik
Ch25h
Asprv1
Naa20
AC168977.1
Apobec1
Mthfr
Tapbpl
Eif2ak2
Relative expression value
(Log2, normalized to mean expression in uninfected animals)
-1
-0.38
0.38
2.5
6
