# Supplementary material for: Natural Killer Cell Sensing of Infected Cells Compensates for MyD88 Deficiency but Not IFN-I Activity in Resistance to Mouse Cytomegalovirus
Source: PLoS Pathog. 2015 May 8;11(5):e1004897. doi: 10.1371/journal.ppat.1004897 (PMC4425567; doi:10.1371/journal.ppat.1004897)
Supplement: S1 File — (DOCX) [file ppat.1004897.s008.docx]

**Supplemental Materials and Methods**

**Mice and *In Vivo* Treatments**

BALB/cByJ mice, referred to as BALB/c, were purchased from Charles River Laboratories and housed at the Centre d’Immunologie de Marseille-Luminy (CIML), Marseille, France. BALB/c Ly49H^+^ (*C.B6-Klra8^Cmv1-r^/UwaJ*) mice were obtained from the Jackson Laboratory [[1](#_ENREF_1)]. BALB/c MyD88^-/-^ (*C.B6.129P2-Myd88^tm1Aki^*) and BALB/c IFNAR^-/-^ mice (*C.B6.129S2-ifnar1^tm1agt^*) were obtained by 10 backcrosses on BALB/c mice of *B6.129-Myd88^tm1Aki^* [[2](#_ENREF_2),[3](#_ENREF_3)] and *B6.129S2-Ifnar1^tm1agt^* [[4](#_ENREF_4),[5](#_ENREF_5)] animals, respectively. BALB/c TLR9^-/-^ mice (*C.129P2-TLR9tm1Aki*) were obtained by 10 backcrosses on BALB/c of *B6.129P2-TLR9tm1Aki* animals [[6](#_ENREF_6)]. BALB/c Ly49H^+^, BALB/c MyD88^-/-^ and BALB/c IFNAR^-/-^ mice, all on BALB/c genetic background, were crossed to generate other mouse strains (BALB/c Ly49H^+^MyD88^-/-^ and BALB/c Ly49H^+^IFNAR^−/−^). Cell depletion efficiency was assessed by flow cytometry on isolated splenocytes, with NK cells identified as NKp46^+^TCRβ^-^ cells, CD8 T cells as CD8α^+^TCRβ^+^ cells and pDCs as CD11c^int^SiglecH^+^ cells, in order to avoid any problem of epitope masking by the depleting antibodies.

**Antibodies and flow cytometry**

The following antibodies were used : B220-APC (RA3-6B2), CD11b-PerCP (M1/70), CD11c-A700 (HL3), CD19-APC (6D5), CD19-FITC (1D3), CD19-PE (1D3), CD3ε-PE (145-2c11), CD4-PE (RM4-5), CD4-PerCP (RM4-5), CD8α-PB (53-6.7), CD8α-PE (53-6.7), CD8α-PerCP (53-6.7), CD8β (H35-17.2), DX5-APC (DX5), Granzyme B-APC (GB11), IFN-β-FITC (RMMB1), IFN-γ-A700 (XMG1.2), IL-12-APC (C15-6), Ki67-PB (B56), Ly49H-Biot (3D10), MHC Class I-PE (H2-Dd 34-5-8S), MHC class II-PE (I-Ad ams32.1), NKp46-PE (29A1.4), CD169-FITC (Moma1), SiglecH-PB (551), Streptavidine-PECy7 (BD Biosciences), TCRβ-APC (H57-597), TCRβ-FITC (H57-597), TCRβ-PE-Cy7 (H57-597), Purified Bst2 (120G8), Purified CD8β (H35-17.2), Purified NK1.1 (PK136), purified polyclonal rat IgG (Jackson ImmunoResearch). Antibodies were purchased from BD biosciences, eBioscience and Biolegend. Extracellular staining was performed at 4°C in PBS, EDTA 2mM, SVF2%, and intracellular staining with a Cytofix/Cytoperm™ Fixation/Permeabilization Solution Kit from BD biosciences.

**Histology**

Freshly extracted spleens were harvested and embedded in optimal cutting temperature (OCT) freezing media (Sakura Finetek). 8 µm frozen spleen sections were cut with a Leica CM3050 S cryostat and fixed in cold acetone for 10 min before saturation with PBS 2% BSA during 30 min. Stainings were performed in PBS by 1 hr incubations with the following antibodies: B220-APC (BD Bioscience, 1:100), CD169-FITC (Serotec, 1:100), CD4-PE and CD8α-PE (BD Bioscience, 1:100). Sections were mounted with Prolong Gold anti-fade reagent (Invitrogen) and photographed with a LSM-510 Carl Zeiss confocal microscope.

**qPCR primers**

Primers were as follows: mouse *Hprt*, 5′-CTGATAAAATCTACAGTCATAGGAATGGA-3′ and 5′-GGCCCTCTGTGTGCTCAAG-3′; mouse *Mx1*, 5′-AGACTTGCTCTTTCTGAAAAGC-3′ and 5′-GACCATAGGGGTCTTGACCAA-3′; mouse *Ifnb1*, 5′-CAGTTTTGGAAGTTTCTGGTAA-3′ and 5′-GGTGGTCCGAGCAGAGATCTT-3′; mouse *Isg15*, 5′-TGGAAAGGGTAAGACCGTCCT-3′ and 5′-GGTGTCCGTGACTAACTCCAT-3′; mouse *Irf7*, 5′-CTTCCCTATTTTCCGTGGCTG-3′ and 5′-TCCAGTTGATCCGCATAAGGT-3′; and MCMV *Ie1*, 5′-GTCGCTGTTATCATTCCCCAC-3′ and 5′-GAGTCGGAACCGAAACCGT-3′.

**Supplemental references**

1. Scalzo AA, Brown MG, Chu DT, Heusel JW, Yokoyama WM, et al. (1999) Development of intra-natural killer complex (NKC) recombinant and congenic mouse strains for mapping and functional analysis of NK cell regulatory loci. Immunogenetics 49: 238-241.

2. Adachi O, Kawai T, Takeda K, Matsumoto M, Tsutsui H, et al. (1998) Targeted disruption of the MyD88 gene results in loss of IL-1- and IL-18-mediated function. Immunity 9: 143-150.

3. Zucchini N, Bessou G, Traub S, Robbins SH, Uematsu S, et al. (2008) Cutting edge: Overlapping functions of TLR7 and TLR9 for innate defense against a herpesvirus infection. Journal of immunology 180: 5799-5803.

4. Strobl B, Bubic I, Bruns U, Steinborn R, Lajko R, et al. (2005) Novel functions of tyrosine kinase 2 in the antiviral defense against murine cytomegalovirus. Journal of immunology 175: 4000-4008.

5. Baranek T, Manh TP, Alexandre Y, Maqbool MA, Cabeza JZ, et al. (2012) Differential responses of immune cells to type I interferon contribute to host resistance to viral infection. Cell host & microbe 12: 571-584.

6. Hemmi H, Takeuchi O, Kawai T, Kaisho T, Sato S, et al. (2000) A Toll-like receptor recognizes bacterial DNA. Nature 408: 740-745.
